# MANF is essential for astrocyte survival in the monkey brain

**DOI:** 10.1093/procel/pwag031

**Published:** 2026-05-03

**Authors:** Tingting Xing, Laiqiang Chen, Xuezhi Duan, Yiran Zhang, Yiyang Qin, Jinpan Lin, Eshu Ruan, Tingting Guo, Changcheng Ye, Jiating He, Suien Xie, Jiawei Zhang, Xichen Song, Wei Huang, Bang Li, Weili Yang, Shihua Li, Xiao-Jiang Li, Su Yang

**Affiliations:** State Key Laboratory of Bioactive Molecules and Druggability Assessment, Guangdong Basic Research Center of Excellence for Natural Bioactive Molecules and Discovery of Innovative Drugs, Guangdong-Hong Kong-Macao Institute of CNS Regeneration, Jinan University, Guangzhou 510632, China; Guangdong Provincial Key Laboratory of Non-human Primate Research, Jinan University, Guangzhou 510632, China; Guangdong Provincial Key Laboratory of Non-human Primate Research, Jinan University, Guangzhou 510632, China; Department of Neurology, The First Affiliated Hospital of Jinan University, Jinan University, Guangzhou 510632, China; Guangdong Provincial Key Laboratory of Non-human Primate Research, Jinan University, Guangzhou 510632, China; Guangdong Provincial Key Laboratory of Non-human Primate Research, Jinan University, Guangzhou 510632, China; Guangdong Provincial Key Laboratory of Non-human Primate Research, Jinan University, Guangzhou 510632, China; Department of Neurology, The First Affiliated Hospital of Jinan University, Jinan University, Guangzhou 510632, China; Guangdong Provincial Key Laboratory of Non-human Primate Research, Jinan University, Guangzhou 510632, China; Guangdong Provincial Key Laboratory of Non-human Primate Research, Jinan University, Guangzhou 510632, China; Department of Neurology, The First Affiliated Hospital of Jinan University, Jinan University, Guangzhou 510632, China; Guangdong Provincial Key Laboratory of Non-human Primate Research, Jinan University, Guangzhou 510632, China; Guangdong Provincial Key Laboratory of Non-human Primate Research, Jinan University, Guangzhou 510632, China; Guangdong Provincial Key Laboratory of Non-human Primate Research, Jinan University, Guangzhou 510632, China; Guangdong Provincial Key Laboratory of Non-human Primate Research, Jinan University, Guangzhou 510632, China; Guangdong Provincial Key Laboratory of Non-human Primate Research, Jinan University, Guangzhou 510632, China; Guangdong Provincial Key Laboratory of Non-human Primate Research, Jinan University, Guangzhou 510632, China; Guangdong Provincial Key Laboratory of Non-human Primate Research, Jinan University, Guangzhou 510632, China; Guangdong Provincial Key Laboratory of Non-human Primate Research, Jinan University, Guangzhou 510632, China; State Key Laboratory of Bioactive Molecules and Druggability Assessment, Guangdong Basic Research Center of Excellence for Natural Bioactive Molecules and Discovery of Innovative Drugs, Guangdong-Hong Kong-Macao Institute of CNS Regeneration, Jinan University, Guangzhou 510632, China; Guangdong Provincial Key Laboratory of Non-human Primate Research, Jinan University, Guangzhou 510632, China; State Key Laboratory of Bioactive Molecules and Druggability Assessment, Guangdong Basic Research Center of Excellence for Natural Bioactive Molecules and Discovery of Innovative Drugs, Guangdong-Hong Kong-Macao Institute of CNS Regeneration, Jinan University, Guangzhou 510632, China; Guangdong Provincial Key Laboratory of Non-human Primate Research, Jinan University, Guangzhou 510632, China; State Key Laboratory of Bioactive Molecules and Druggability Assessment, Guangdong Basic Research Center of Excellence for Natural Bioactive Molecules and Discovery of Innovative Drugs, Guangdong-Hong Kong-Macao Institute of CNS Regeneration, Jinan University, Guangzhou 510632, China; Guangdong Provincial Key Laboratory of Non-human Primate Research, Jinan University, Guangzhou 510632, China; Lingang Laboratory, Shanghai 200031, China; State Key Laboratory of Bioactive Molecules and Druggability Assessment, Guangdong Basic Research Center of Excellence for Natural Bioactive Molecules and Discovery of Innovative Drugs, Guangdong-Hong Kong-Macao Institute of CNS Regeneration, Jinan University, Guangzhou 510632, China; Guangdong Provincial Key Laboratory of Non-human Primate Research, Jinan University, Guangzhou 510632, China

**Dear Editor,** 

Mesencephalic astrocyte-derived neurotrophic factor (MANF) is classified as a non-canonical neurotrophic factor due to its unique structural and functional characteristics. Administration of recombinant MANF protein has demonstrated neuroprotective benefits in models of Parkinson’s disease (PD) and retinal degeneration (Airavaara et al., 2009; Neves et al., 2016). Intracellularly, MANF is predominantly localized to the endoplasmic reticulum (ER), where its expression is upregulated in response to ER stress (Apostolou et al., 2008). Germline deletion of *Manf* in mice leads to growth retardation due to the loss of pancreatic β cells, yet these mice do not exhibit obvious neuronal phenotypes (Lindahl et al., 2014). Moreover, two independent studies have reported that conditional *Manf* knockout mice lacking MANF expression in the brain have normal behavioral performances and no significant neuronal loss (Pakarinen et al., 2020; Wang et al., 2021). In humans, however, homozygous loss-of-function mutations in the *MANF* gene result in neurodevelopmental defects, including intellectual disability and microcephaly, in addition to diabetes (Montaser et al., 2021; Yavarna et al., 2015). Non-human primates exhibit a greater resemblance in both structure and function to the human brain. Consequently, non-human primates serve as an excellent model for investigating the functions of specific proteins within the central nervous system. Here, we utilized CRISPR/Cas9 genome editing tools to knock down MANF expression in the brains of adult mice and cynomolgus monkeys (*Macaca fascicularis*) and examined the impact on neuronal and glial survival.

We examined MANF expression in the cortex, striatum, cerebellum, and hippocampus of adult WT mice (8-month-old) and monkeys (5-year-old). Western blotting showed that in mice, MANF protein levels were highest in the cortex, whereas in monkeys, the hippocampus exhibited the strongest expression (Fig. S1A–C). qRT-PCR revealed comparable *MANF*/*Manf* mRNA levels across brain regions in both species (Fig. S1D). Immunohistochemistry further confirmed elevated MANF expression in the mouse cortex and monkey hippocampus (Fig. S1E and S1F). Double immunofluorescence staining demonstrated that MANF is also widely distributed in neurons (NeuN^+^), astrocytes (GFAP^+^), and microglia (Iba1^+^) in monkey brains (Fig. S1G and S1H).

The absence of apparent neuronal phenotypes in *Manf* knockout mice could be due to genetic compensation. To acutely reduce MANF expression, we utilized CRISPR/Cas9 genome editing tools. We designed gRNAs targeting mouse *Manf* and monkey *MANF* (Figs. 1A and S2A–D) and packaged them into adeno-associated viruses (AAVs, serotype: AAV-Mac). The selected gRNAs exhibit minimal off-target activity, as sequencing of the three most likely off-target sites revealed no mutations induced by CRISPR/Cas9 (Fig. S2E). We first knocked down MANF in mouse primary neurons by adding AAVs at 2 days *in vitro* (DIV 2) and collecting cells 5 days later. Western blotting confirmed a strong reduction in MANF protein, while levels of NeuN, β3-tubulin, and PSD95 remained unchanged (Fig. S3A and S3B). Immunofluorescence also showed decreased MANF staining in GFP-positive neurons infected with AAV-Manf-gRNA (Fig. S3C and S3D), but both pMAP2 intensity and neurite complexity were comparable to controls (Fig. S3E–G). We next examined whether MANF reduction affects astrocytes. Mouse primary astrocytes were transduced with AAV-Manf-gRNA or AAV-Ctrl-gRNA and collected 5 days later. Western blotting confirmed a marked decrease in MANF but no changes in the astrocyte markers ALDH1L1, SOX9, or GFAP (Fig. S4A and S4B). Immunofluorescence also showed reduced MANF staining (Fig. S4C and S4D), whereas SOX9 and GFAP intensities remained comparable between groups (Fig. S4E–H). To assess the role of MANF in the adult brain, we stereotaxically injected AAV-Cas9/AAV-Manf-gRNA or AAV-Cas9/AAV-Ctrl-gRNA into the hippocampus of 3-month-old WT mice. One month later, MANF protein levels were markedly reduced, while neuronal (NeuN, PSD95) and astrocytic (GFAP, S100β) markers remained unchanged (Fig. S5A and S5B). Immunofluorescence confirmed strong MANF loss in GFP-positive regions without detectable changes in NeuN or GFAP staining (Fig. S5C–H). We also knocked down MANF in the substantia nigra (Fig. S6A and S6B), but TH staining of dopaminergic neurons was unaffected (Fig. S6C and S6D).

**Figure 1.**
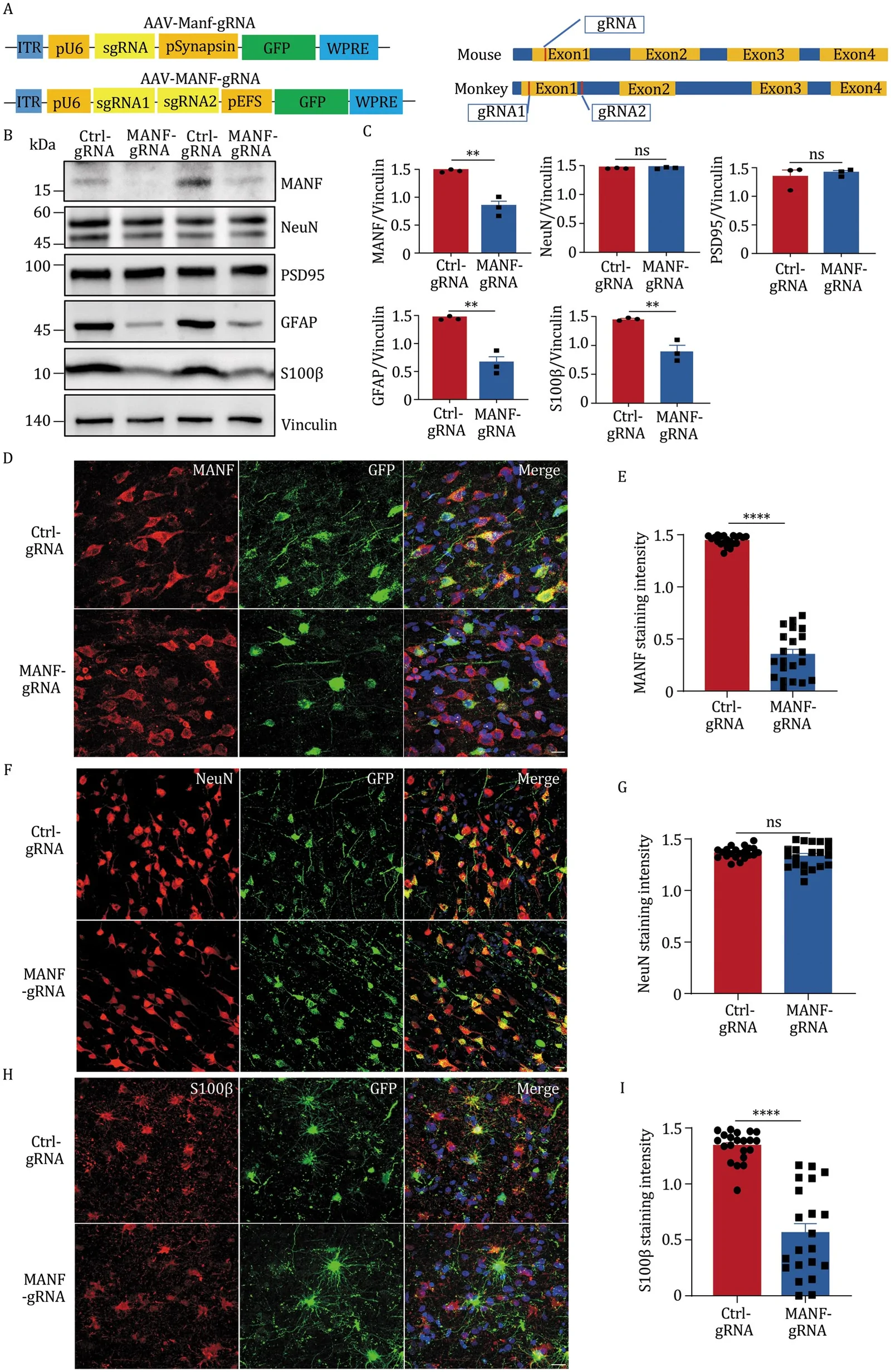
**Knockdown of MANF led to a reduction of astrocytes in the monkey brain**. (A) Schematic representation of AAV-Manf-gRNA and AAV-MANF-gRNA constructs. (B) Western blotting analysis of MANF, NeuN, PSD95, GFAP, and S100β in the cortex of WT monkeys injected with AAV-Cas9/AAV-Ctrl-gRNA or AAV-Cas9/AAV-MANF-gRNA. Vinculin was used as a loading control. (C) Quantification of western blotting results in Fig. 1B (*n *= 3, two-tailed Student’s *t*-test). (D) Immunofluorescent staining of MANF in the brain of WT monkeys injected with AAV-Cas9/AAV-Ctrl-gRNA or AAV-Cas9/AAV-MANF-gRNA. GFP fluorescence reflected the AAV-infected cells (scale bar: 20 μm). (E) Quantification of MANF staining intensity in GFP-positive cells (*n *= 21 from three monkeys, two-tailed Student’s t-test). (F) Immunofluorescent staining of NeuN in the brain of a WT monkey injected with AAV-Cas9/AAV-Ctrl-gRNA or AAV-Cas9/AAV-MANF-gRNA (scale bar: 20 μm). (G) Quantification of NeuN staining intensity in Fig. 1F (*n *= 21 from three monkeys, two-tailed Student’s *t*-test). (H) Immunofluorescent staining of S100β in the brain of a WT monkey injected with AAV-Cas9/AAV-Ctrl-gRNA or AAV-Cas9/AAV-MANF-gRNA (scale bar, 20 μm). (I) Quantification of S100β staining intensity in Fig. 1H (*n *= 21 from three monkeys, two-tailed Student’s *t*-test). ns, non-significant, **P < 0.01, ***P < 0.001, ****P < 0.0001. Data are represented as mean ± SEM.

We next reduced MANF expression in the adult monkey hippocampus using stereotaxic delivery of AAV-Cas9/AAV-MANF-gRNA or AAV-Cas9/AAV-Ctrl-gRNA. One month later, western blotting confirmed a strong decrease in MANF, with NeuN and PSD95 levels unchanged. In contrast, GFAP and S100β were significantly reduced (Fig. 1B and 1C). Immunofluorescence showed markedly decreased MANF staining in GFP-positive cells (Fig. 1D and 1E). While NeuN staining in GFP-positive neurons remained unaffected (Fig. 1F and 1G), S100β intensity was significantly reduced in GFP-positive astrocytes (Fig. 1H and 1I). Additional astrocyte markers, including GFAP and SOX9, showed similar reductions (Fig. S7A–D). We also used Sholl analysis to assess astrocyte process complexity and found a significant reduction following MANF knockdown (Fig. S7E). Together, these results suggest that MANF depletion preferentially affects astrocytes in the monkey brain, although we cannot rule out the possibility that the astrocyte loss is an indirect consequence of primary neuronal damage.

We extended the analysis to an *in vitro* monkey system. Due to technical difficulties in culturing primary fetal monkey neurons, we focused on culturing astrocytes (Fig. S8A and S8B) to study the effects of MANF. Given the low transduction efficiency of AAVs in monkey astrocytes, we adopted a more effective approach using siRNA electroporation. Four siRNAs targeting monkey MANF (462, 507, 549, and 679) produced similar knockdown efficiency in COS7 cells (Fig. S8C and S8D). Three of them (462, 507, and 679) were then pooled to suppress MANF expression in primary monkey astrocytes and to minimize potential off-target effects from individual siRNAs (Fig. S8E). Three days after siRNA electroporation, astrocytes with MANF knockdown exhibited significantly higher levels of TUNEL staining (Fig. S9A and S9B) and lower MTT scores (Fig. S9C), indicating that MANF reduction affects monkey astrocyte survival. Therefore, we collected the astrocytes for RNAseq. *MANF* knockdown produced 401 differential expression genes (DEGs): 118 upregulated and 283 downregulated (Fig. 2A and 2B). GO analysis revealed enrichment of metabolic pathways among upregulated genes, and axon development, axonogenesis and gliogenesis pathways among downregulated genes (Fig. 2C). Because gliogenesis is critical for astrocyte regenerative capacity, we focused on this pathway. Multiple glial regeneration-related markers were significantly reduced in MANF-siRNA samples (Fig. S9D). qRT-PCR of selected DEGs confirmed the RNAseq results (Fig. S9E). Among the downregulated genes, ADORA2A promotes astrocyte activation and proliferation (Paiva et al., 2019). Leukemia inhibitory factor (LIF) is an essential cytokine for astrocyte differentiation, which signals through the JAK/STAT3 pathway to support cell growth and survival (Koblar et al., 1998; Nakashima et al., 1999). Their reduced expression is consistent with the astrocyte loss observed after MANF depletion.

**Figure 2.**
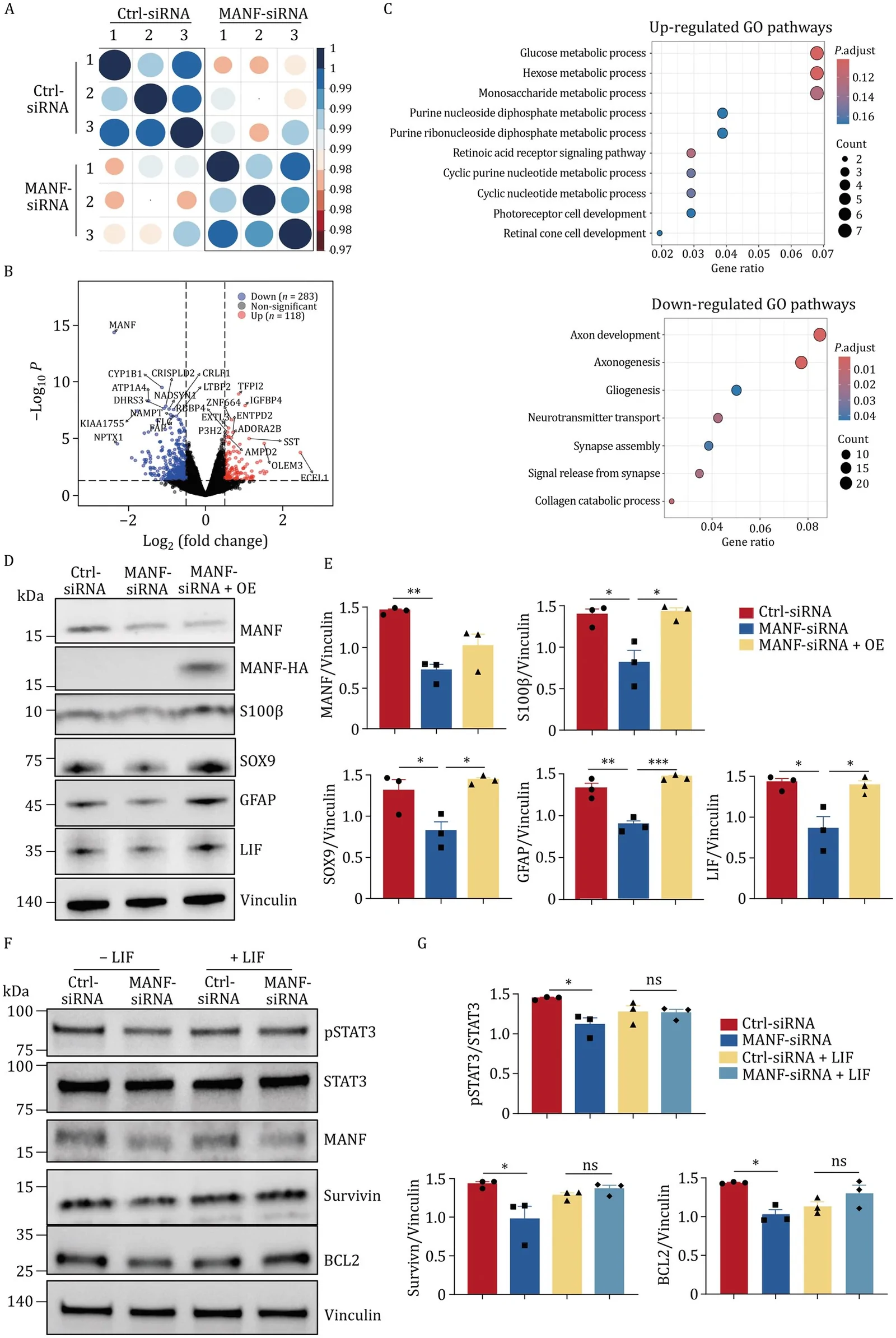
**MANF depletion impacts LIF signaling pathway in monkey astrocytes**. (A) Correlation plots showing the overall transcriptional similarity among different samples of monkey primary astrocytes electroporated with Ctrl-siRNA or MANF-siRNA (*n *= 3 for each group). (B) Volcano plots showing significantly upregulated (*n *= 118) and downregulated (*n *= 283) genes when comparing the monkey primary astrocytes electroporated with Ctrl-siRNA or MANF-siRNA. (C) GO enrichment analysis of the biological pathways that are significantly upregulated or downregulated. (D) Western blotting analysis of MANF, HA, S100β, SOX9, GFAP, and LIF in the monkey primary astrocytes electroporated with Ctrl-siRNA, MANF-siRNA, or co-electroporated with MANF-siRNA and siRNA-resistant MANF (OE). Vinculin was used as a loading control. (E) Quantification of western blotting results in Fig. 2D (*n *= 3, one-way ANOVA with Tukey’s post-tests). (F) Monkey primary astrocytes electroporated with Ctrl-siRNA or MANF-siRNA have been treated with or without recombinant LIF. Western blot analysis was conducted to assess the expression of MANF, pSTAT3, STAT3, Survivin, and BCL2. (G) Quantification of western blotting results in Fig. 2F (*n *= 3, one-way ANOVA with Tukey’s post-tests). ns, non-significant, *P < 0.05, **P < 0.01, ***P < 0.001. Data are represented as mean ± SEM.

To further evaluate MANF function in monkey astrocytes, we analyzed several astrocyte markers by western blotting. MANF knockdown caused significant reductions in GFAP, S100β, SOX9, and LIF (Fig. 2D and 2E). We further generated a mutant form of MANF that is resistant to siRNA-mediated knockdown. The mutant MANF plasmid contains an HA tag at the C-terminus, which makes it unrecognizable by the MANF antibody but enables detection with an HA antibody. Expression of the mutant MANF significantly rescued the reduction of GFAP, S100β, SOX9, and LIF in monkey astrocytes treated with MANF-siRNA (Fig. 2D and 2E). In MANF-depleted monkey brain tissues, similar decreases in LIF and ADORA2A were observed (Fig. S9F and S9G). These findings suggested that boosting LIF signaling might counteract MANF-loss-induced astrocyte damage. Indeed, treatment of monkey astrocytes with 100 ng/mL recombinant human LIF rapidly increased pSTAT3 levels (Fig. S9H and S9I). With daily LIF treatment for 72 h, MANF-deficient astrocytes showed elevated pSTAT3 and restored expression of the anti-apoptotic markers Survivin and BCL2, both of which were reduced following MANF depletion (Fig. 2F and 2G). Therefore, MANF knockdown promotes apoptosis in monkey astrocytes, which can be rescued by LIF supplementation. Repeating these experiments in mouse astrocytes showed no changes in LIF, ADORA2A, or pSTAT3 after MANF knockdown (Fig. S10A and S10B).

MANF is broadly expressed in the central nervous system. Although it has long been recognized as a neuroprotective factor, its cell-type-specific roles in the brain have remained incompletely understood. In knockout mice, which lack MANF expression in the brain, no overt neurodegeneration or noticeable neurological deficits were found (Pakarinen et al., 2020; Wang et al., 2021). These observations contrast sharply with the severe neurodevelopmental abnormalities seen in humans with MANF deficiency, who present with intellectual disability and microcephaly (Montaser et al., 2021; Yavarna et al., 2015).

In this study, we systematically compared the expression patterns and functional roles of MANF in rodent and primate brains, uncovering important species-specific differences. Notably, we found that acute MANF reduction leads to different cellular consequences in mice and monkeys: while MANF depletion does not affect neuron or astrocyte survival in mice, it leads to astrocyte loss in monkeys. Given that disruptions in glial cell function are known to contribute to microcephaly (Quincozes-Santos et al., 2023), our findings offer a potential explanation for the microcephaly observed in humans with MANF deletion.

We extended our study to primary monkey astrocytes. An important caveat is that the function of MANF may differ between adult brain and fetal astrocytes. Nevertheless, the astrocyte culture enabled us to investigate the effects of MANF knockdown using a high-throughput sequencing approach. Indeed, our results demonstrate that MANF knockdown leads to transcriptional alterations, including the downregulation of genes involved in gliogenesis. Among these changes, the LIF-STAT3 pathway emerged as a key candidate. LIF is a cytokine secreted by astrocytes, and activation of its downstream effector STAT3 plays a central role in cellular protection (Samad et al., 2025). Our results demonstrate that MANF knockdown led to reductions in LIF expression, decreased STAT3 activation, and diminished levels of anti-apoptotic proteins such as BCL2 and Survivin. LIF is well known to function as a paracrine factor that supports neuronal and oligodendrocyte survival (Linnerbauer and Rothhammer, 2020; Ratti et al., 2021). Our findings further suggest that LIF also acts, as an autocrine manner to mediate anti-apoptotic effects on astrocytes themselves. Nonetheless, the mechanism by which LIF reduction occurs in monkey astrocytes but not in mouse astrocytes remains to be determined. Given that ER stress primarily alters gene transcription through the Unfolded Protein Response (UPR) (Chen et al., 2023), it is likely that MANF depletion in monkey astrocyte activates certain UPR-responsive elements, such as PERK-dependent repression program, that repress LIF transcription in a primate-specific manner.

Together, our findings uncover a previously unappreciated primate-specific role of MANF in regulating astrocyte survival through the LIF-STAT3 pathway. The divergent responses to MANF depletion between rodents and primates underscore the importance of using non-human primates to investigate MANF neurobiology and to evaluate therapeutic strategies targeting MANF-related pathways. Moreover, given the central role of astrocytes in metabolic support, synapse maintenance, and neuroprotection, our results suggest that therapeutic strategies aimed at augmenting MANF or LIF signaling may hold promise for conditions characterized by astrocyte dysfunction, including neurodevelopmental or neurodegenerative diseases.

## Supplementary Material

pwag031_Supplementary_Data

## Footnotes

This work was supported by the National Key R&D Program of China (2021YFA0805200 to S.Y.), Fundamental Research Funds for the Central Universities (2026KQZX101 to S.Y.), the National Natural Science Foundation of China (82471437 to S.Y., 82394422 to X.J.L., 82371874 to X.J.L., 32541012 to X.J.L., 82271902 to S.H.L., U24A6013 to S.H.L.), Department of Science and Technology of Guangdong Province (2025A1515011184 to S.Y., 2021ZT09Y007 to X.J.L., 2019QN01Y417 to X.J.L., 2026A1515011170 to Y.Y.Q.). Su Yang gratefully acknowledges the support of K. C. Wong Education Foundation.

The authors declare no competing interests. All authors give their consent for publication. The authors used AI-assisted language refinement to improve the readability of the manuscript.

Mouse-related procedures were submitted to the IACUC of Jinan University under application number 12806 and approved under approval number IACUC-20210220-06. Monkey-related procedures were approved by the IACUC of Guangdong Landau Biotechnology Co., Ltd. (approval number: LDACU20201216-02).

The data required to evaluate the conclusions are provided in the paper and the Supplemental materials. Research materials are available from the corresponding authors upon request.

S.Y. conceived the project and designed research; T.X., L.C., X.D., Y.Z., Y.Q., J.L., E.R., T.G., C.Y., S.X., J.Z., X.S., and W.H. performed research; B.L., W.Y., S.L., and X.J.L. provided key research samples and experimental techniques; T.X. and S.Y. analyzed results and wrote the manuscript; S.L. and X.J.L. edited the manuscript.
