# Supplementary material for: MANF is essential for astrocyte survival in the monkey brain
Source: Protein Cell. 2026 May 3;17(11):1060–5. doi: 10.1093/procel/pwag031 (PMC13721127; doi:10.1093/procel/pwag031)
Supplement: Supplementary file 1 [file pwag031_supplementary_data.zip › Supplemental_Material.pdf]

MANF is essential for astrocyte survival in the monkey brain.

Tingting Xing[1, 2, #], Laiqiang Chen[2, 3, #], Xuezhi Duan[2, #], Yiran Zhang[2], Yiyang Qin[2, 3], Jinpan Lin[2], Eshu Ruan[2, 3], Tingting Guo[2], Changcheng Ye[2], Jiating He[2], Suien Xie[2], Jiawei Zhang[2], Xichen Song[2], Wei Huang[2], Bang Li[2], Weili Yang[1, 2], Shihua Li[1, 2], Xiao-Jiang Li[1, 2, 4 *], Su Yang[1, 2, *]

[1]State Key Laboratory of Bioactive Molecules and Druggability Assessment, Guangdong Basic Research Center of Excellence for Natural Bioactive Molecules and Discovery of Innovative Drugs, Guangdong-Hong Kong-Macau Institute of CNS Regeneration, Jinan University, Guangzhou, China.

[2]Guangdong Provincial Key Laboratory of Non-human Primate Research, Jinan University, Guangzhou, China.

[3]Department of Neurology, The First Affiliated Hospital of Jinan University, Jinan University, Guangzhou, China.

[4]Lingang Laboratory, Shanghai, China

***Correspondence:** Xiao-Jiang Li (xjli33@jnu.edu.cn), Su Yang (syang33@jnu.edu.cn)

#Co-first authors: Tingting Xing, Laiqiang Chen, Xuezhi Duan

Running title: MANF supports astrocyte survival in monkeys

Key words: MANF, non-human primate, neuron, astrocyte, LIF

Materials and methods

Animals

Wild-type C57BL6J mice were purchased from Guangdong Medical Laboratory Animal Center (Guangzhou, China). The mice were maintained in a 12-h light/dark cycle in the Division of Animal Resources of Jinan University. The monkeys were maintained at Guangdong Landau Biotechnology Co. Ltd., which is an Association for Assessment and Accreditation of Laboratory Animal Care (AAALAC) accredited facility. Mouse-related procedures were submitted to the IACUC of Jinan University under application number 12806 and approved under approval number IACUC-20210220-06. Monkey-related procedures were approved by the IACUC of Guangdong Landau Biotechnology Co. Ltd. (approval number: LDACU20201216-02). The sex of the animals was not considered when performing experiments.

Cell culture

COS7 and N2A cells were purchased from ATAC (CRL-1651, and CCL-131) and cultured in Dulbecco's Modified Eagle Medium (Gibco, C11995500CP) containing 10% fetal bovine serum (Gibco, 10091148), penicillin and streptomycin (100 U/ml; Gibco, 15140163). The culture medium was replaced every 48 hours. For transient transfection, cells at 70–80% confluency were transfected with plasmids or siRNAs using Lipofectamine 3000 (L3000001, Invitrogen) as recommended by the manufacturer. Cells were harvested 48 hours post-transfection.

Primary neuron and astrocyte culture

Cortical tissues were dissected from monkey fetuses at 90–100 days of gestation. The tissues

were minced into uniform 1 mm^3 chunks using sterile scissors and subjected to 1 mg/ml papain (Worthington, LS003119) digestion in PBS buffer containing 1× DNase at 37°C for 15 minutes. During incubation, the tissues were gently triturated multiple times using a 1 ml pipette tip to facilitate dissociation into single cells. The suspension was centrifuged at 300×*g* for 5 minutes. Culture dishes were pre-coated with 0.1 mg/mL poly-D-lysine (BBI, A600751-0025) for at least 1 hour at room temperature. Cells were seeded at a density of 1×10^5 cells/mL in complete culture medium. The culture medium consisted of DMEM supplemented with 10% FBS and 100 U/mL penicillin-streptomycin Upon reaching confluence in 10 cm culture dishes, the cells were washed and digested with 0.05% trypsin. The Lonza Electroporation Kit was used for transfection. Following electroporation, the cells were seeded into 6-well plates and maintained under standard culture conditions. The medium was replaced 24 hours post-transfection, and cells were harvested 72 hours after transfection for subsequent protein and RNA analysis. For the recombinant human LIF (MCE, HY-P73276) protein treatment, LIF was added to the culture medium at a concentration of 50 ng/ml at the specified stages. The medium was replaced every 24 hours with fresh medium containing the same concentration of LIF until the experiment was complete.

Primary mouse neurons and astrocytes were cultured from the cortex of newborn mice on day 1 postnatal for neurons and day 2 or 3 postnatal for astrocytes, followed by digestion. Primary neurons were cultured in Neurobasal medium, while astrocytes were maintained in complete DMEM medium. AAVs were added to mouse primary neurons on the second day post-seeding. After that, the medium was changed every two days until day six, when the cells were

harvested. AAVs were added to mouse primary astrocytes when they reached 70% confluence after passage and harvested five days later.

Western blotting

Fresh brain tissues or cell samples were lysed using ice-cold RIPA buffer supplemented with a protease inhibitor cocktail (Mei5bio, MF182-plus-10) and phosphatase inhibitors (Sigma, S7920 and S6508). The lysates were subsequently sonicated 5-6 times and centrifuged at 12,000 × *g* for 15 minutes at 4°C to pellet insoluble debris. SDS-PAGE (CWBIO, CW8205S) was conducted and the proteins in the gel were transferred to a nitrocellulose membrane. The membranes were blocked with 5% skim milk for 1 hour at room temperature and subsequently washed three times with PBS. Following washing, the blots were incubated overnight at 4°C with primary antibodies. The next day, after three additional washes with PBS, the membranes were incubated with HRP-conjugated secondary antibodies diluted in 5% skim milk prepared in TBST for 1 hour at room temperature. The blots were then washed three times in TBST and developed using ECL Prime (Millipore, WBKLS0500). The images were acquired digitally using Clinx ChemiScope 6300.

Primary antibodies

MANF (LSBio, B2688; Abcam, ab67271; EM572, custom-made); NeuN (Millipore, MAB377; Abcam, ab177487; Sigma, ABN90); GFAP (Abcam, ab7260; Santa Cruz, 33673); SOX9 (Sigma, AB5535); S100beta (Abcam, ab52642); PSD95 (Abcam, ab238135); Cas9 (Millipore, MAC133); β3-tubulin (BioLegend, 801202); Vinculin (Millipore, MAB3574; Proteintech, 26520-

1-AP); pMAP2 (Abcam, ab96378); ADORA2A (Santa Cruz, 32261); STAT3 (Abmart, T55292F); pSTAT3 (Abmart, T56566F; Cell Signaling, 9145S); Survivin (Immunoway, PT0343R); LIF (Abclonal, A1288; Proteintech, 26757-1-AP); BCL2 (HUABIO, ET1702-53); GFP (Emarbio, EM33012); TH (Abcam,ab112); HA (Proteintech, 51064-2-AP); GAPDH (Proteintech 60004-1-Ig); Iba1 (Oasis Biofarm, OB-PGP049-01); ALDH1L1 (Gentex, GTX84894).

siRNAs

The siRNAs utilized in this study were acquired from GenePharma. The siRNA sequences are listed as follows: Ctrl-siRNA, sense: 5’-UUC UCC GAA CGU GUC ACG UTT-3’, antisense, 5’-ACG UGA CAC GUU CGG AGA ATT-3’; MANF-siRNA462, sense, 5’-GAA GAU CUG UGA GAA GCU UTT-3’, antisense, 5’-AAG CUU CUC ACA GAU CUU CTT-3’; MANF-siRNA507, sense, 5’-GCU AAA GUA UGA CAA GCA GTT-3’, antisense, 5’-CUG CUU GUC AUA CUU UAG CTT-3’; MANF-siRNA679, sense, 5’-GCC AGU GCA CGG ACU GAU UTT-3’, antisense, 5’-AAU CAG UCC GUG CAC UGG CTT-3’.

Quantitative real-time PCR

The TRIzol (Invitrogen) reagent was utilized to extract the RNA from the cells. cDNA synthesis was performed using the PrimeScript RT Reagent Kit (Takara, RR047A) with gDNA Eraser. The QuantiNova™ SYBR Green PCR kit (QIAGEN, 208,052) was utilized for quantitative real-time PCR. The reaction was performed in the CFX Connect Real-Time System (Bio-Rad). Primers used are listed as follows. *ACTIN*, forward: 5’-ACA GAG CCT CGC CTT TGC-3’, reverse: 5’-CAC GAT GGA GGG GAA GAC-3’; *Actin*, forward: 5’-TCA CTG TCC ACC TTC CAG CAG ATG-

3’, reverse: 5’-CTC AGT AAC AGT CCG CCT AGA AGC-3’; *MANF*, forward: 5’-TCT GGC CCA CCA CAT CCC T-3’, reverse: 5’-TCC ACT GTG CTC AGG TCG AT-3’; *Manf*, forward: 5’-CAA GAG GCA AAG AGA ATC GG-3’, reverse: 5’-CAC AGA TCT TTT CCA CAG GG-3’; *LIF*, forward: 5’-GCC ACC CAT GTC ACA ACA AC-3’, reverse: 5’-CCC CCT GGG CTG TGT AAT AG-3’; *ADORA2A*, forward: 5’-AGA TCA TTC GCA GCC ATG TCC T-3’, reverse: 5’-GGC TGA CCT GCT CTC CAT CAC T-3’; *DLL1*, forward: 5’-CAA GGC GAG GGA GCT TTT C-3’, reverse: 5’-GAG CTC CAG ACC TGA CAC A-3’; *LAMC3*, forward: 5’-AGC TGC AGT TTC CTC GGC CCA A-3’, reverse: 5’-GGC CGC GTT TCC TAG CAT CCT C-3’;

Virus packaging

AAV-Manf-gRNA and AAV-Ctrl-gRNA were generated and described in our previous study (Yang et al., 2017). The MANF-gRNA plasmid contains two distinct gRNA sequences: gRNA1 is 5’- GCG CCG TAG CTC CCC ATT GG -3’, and gRNA2 is 5’- TAC CGC TCC CCG TCC GAC GT -3’. The two gRNA sequences were inserted into the ssAAV.U6.sgRNA.EFS.GFP plasmid (purchased from PackGene Biotech). These viral vectors were sent to PackGene Biotech Inc for packaging into AAV-MAC or AAV9 serotypes (1×10^{13} vg/ml).

Stereotaxic injection

For mouse stereotaxic surgery, the mice were anesthetized with continuous 1.5% isoflurane inhalation and fixed on a stereotaxic instrument (RWD, 69100). The hair around the surgical site was removed using disinfected scissors and the skin was sterilized with 70% alcohol. The skin was cut to expose the bregma of the skull, and the injection sites were identified according

to the distance from the bregma: hippocampus, anterior-posterior = -2.5 mm, medial-lateral = ±2.0 mm, dorsal-ventral = -2.0 mm; substantia nigra, anterior-posterior = -3.3 mm, medial-lateral = ±1.5 mm, dorsal-ventral = -4.7 mm. Microdrill was used to make small holes in the skull, and the AAV (1E+13 GC/ml) was injected into the brain using 33G microsyringe (Hamilton. NanoFil-10ul-1) at the speed of 200 nL/min. The microsyringe was left in the injection site for 10 min, and then slowly withdrawn. All post-surgical mice were closely monitored.

For monkey stereotaxic surgery, the monkey was anesthetized by intraperitoneal injection of 0.3-0.5 mg atropine, followed by 10-12 mg ketamine and 15-20 mg pelltobarbitalumnatricum per kg body weight. The monkey was stabilized on a stereotaxic instrument (RWD, 68901). The precise positions of the hippocampus were determined by Magnetic Resonance Imaging (MRI) before injection. A microsyringe was used to deliver 10 µl of viruses into each location. Post-operative care was given by certified veterinarians at Guangdong Landau Biotechnology Co. Ltd. Three male monkeys were used for injection, and they were 5.0, 5.1, and 7.4 years old, respectively.

Immunofluorescent staining

The animals were transcardially perfused with warm 0.9% saline followed by ice-cold 4% paraformaldehyde (PFA) in 0.1 M phosphate buffer (PB). The brains were post-fixed overnight in 4% PFA, then cryoprotected by sequential immersion in 15% and 30% sucrose solutions. The brains were embedded in OCT compound (Sakura, 4583) and sectioned at 30 µm using a cryostat (CryoStar NX50, Thermo Fisher). The brain slices or cells were washed three times in

1xPBS and blocked for 30 min at room temperature with 3% bovine serum albumin and 2% goat serum in PBS containing 0.1% Triton X-100 (Sigma, X100). After blocking, the slices or cells were incubated overnight at 4°C with primary antibodies diluted in the blocking buffer. The following day, the slices or cells were washed three times with PBS and incubated in PBS containing fluorescent secondary antibodies and DAPI for 1 hour at room temperature. Images were acquired using an Olympus FV3000 confocal laser scanning microscope. The staining intensity was measured using ImageJ (NIH) software (version 1.54d). The images were converted to 8-bit grayscale. The “threshold” function was used to subtract background, and the “measure” function was used to quantify the staining intensity. The Sholl analysis plug-in (https://imagej.net/plugins/sholl-analysis) was used to measure process complexity of neurons and astrocytes.

Immunohistochemistry

The brain slices underwent three washes in 1xPBS and then treated in 0.3% hydrogen peroxide for 10 minutes to inhibit endogenous peroxidase activity, They were subsequently washed three additional times with 1X PBS and blocked for 1 hour in PBS containing 3% bovine serum albumin, 2% normal goat serum, and 0.1% Triton X-100, followed by incubation in the blocking buffer containing primary antibodies at 4 °C overnight. On the following day, the Mouse and Rabbit Specific HRP/DAB (ABC) Detection IHC kit (Abcam, ab64264) was used according to the manufacturer's protocol. Zeiss AX10 Axio microscope was used to acquire images.

TUNEL staining and MTT assay

One Step TUNEL Apoptosis Assay Kit (Beyotime, C1089) was used following the manufacturer's protocol. Briefly, the cells were fixed with 4% paraformaldehyde for 30 min at room temperature and washed three times with 1× PBS. The cells were permeabilized with 0.3% Triton X-100 in PBS for 10 min, followed by incubation with the TUNEL detection solution (consisting of 5 μL TdT enzyme and 45 μL fluorescent labeling solution) for 1 h at 37°C in a humidified dark chamber. After three additional washes with 1× PBS, cells were incubated with DAPI solution (Beyotime, C1002) for 5 min at room temperature to label nuclei. MTT assay kit (Solarbio, M1020) was used following the manufacturer's protocol. The absorbance was recorded using a microplate spectrophotometer (EPOCH, BioTek).

RNA sequencing and analysis

Total RNAs from monkey primary astrocytes were extracted using Trizol reagent according to the manufacturer's protocol and subsequently sent to Novogene (Beijing, China) for RNA sequencing. The raw RNA-seq data were mapped and quantified using Salmon (ver 1.9.0) with the Macaca fascicularis 5.0 (macFas5) genome as reference. Quantification files were imported and summarized into count matrix using the tximport package (ver 1.28.0). Differential gene expression analysis was performed using edgeR. Sample-wide expression matrix was used for correlation analysis between samples and visualized with the corrplot package (ver 0.95). The differentially expressed genes (DEGs) between MANF-siRNA and Ctrl-siRNA groups were identified using the threshold of P value < 0.05 and |fold change| > 0.5 with edgeR. Volcano plots were generated using the EnhanceVolcano package to exhibit up-regulated and down-regulated DEGs (ver 1.14.0). The samples expression profiles were clustered and visualized

with ComplexHeatmap (ver 2.13.1).

The up-regulated and down-regulated DEGs were put to Gene Ontology (GO) pathway analysis using ClusterProfiler (ver 4.16.0). To further investigate changes in astrocyte-related genes between MANF-siRNA and Ctrl-siRNA groups, the genes in the pathways of “GO:0060018","GO:0021896”, “GO:0021897”, “GO:0061888”, “GO:0036520”, and “GO:0014002” were used to generate the heatmap plot. All the above analysis were performed on the R (ver 4.6.0) and R studio (ver 2025.09.0). The raw data has been deposited to the BioProject database with the accession number PRJNA1375749.

Off-target analysis for gRNA and siRNA

Potential off-target sites for MANF-gRNA were identified using CRISPOR (https://crispor.gi.ucsc.edu). The top three sites were amplified via PCR and cloned into T5 vector (TransGen Biotech, CT501). At least 10 independent clones were sent for Sanger sequencing. The primers used for PCR are listed below.

Manf-gRNA: *Aunip*, forward 5’- CCC GGC TTC CTT AGC GCA CT-3’, reverse 5’- AGA GGC GGA AGA TGC AGG TGG-3’; *Trim30d*, forward 5’- CC GAG GTC TAA GAG GCG GC-3’, reverse 5’- TGG AAT CAA ACC CTT CCG ACC-3’; *Tbx4*, forward 5’- CTT GTC CCT CAC ACA TGG GG-3’, reverse 5’- ACA AAC AGC CTC AGT CTG GG-3’. MANF-gRNA1: *NUDT5*, forward 5’- CTG GCA TTG CTG ACC TCT CA-3’, reverse 5’- TGC TTA GGG ATG TTG GCC TG-3’; *CPNE6*, forward 5’- GGA TTA CAT CAT GGG TGG CTG, reverse 5’- CCC GAC AGC CCA ACC

ACT A-3’; *CDC23*, 5’-CAT CTT GAC CCT AGA GAA TGC CA-3’, reverse 5’- TGA AGG AGG GCA GTT GCT TT-3’. MANF-gRNA2: *CDKN2AIPNL*, forward 5’- TCA GAG AGC GAG AAG CAA TGG-3’, reverse 5’- GGG CCA ACC ATC TCT TCC TAG-3’; *KIAA1671*, forward 5’- CCA GTA GCC GTT TGT CTT CTC T-3’, reverse 5’- GAA TCC ACC GAC GGG ATG GA-3’; *MLC1*, forward 5’-AGC CCC TGG TTT GTC TCA TGC-3’, reverse 5’- TCC CAT TGG TGG CAG CTG TG-3’.

Potential off-target effects of the designed siRNAs were evaluated using the NCBI Nucleotide BLAST tool (https://blast.ncbi.nlm.nih.gov/Blast.cgi). The top three candidate genes exhibiting the highest degree of sequence similarity to the siRNA targets were selected for quantitative real-time PCR. The primers used are listed below.

MANF 462: *TDRP*, forward 5’- AGC CTC CAA AAC TTG CTC TT-3’, reverse 5’- CTC GAC CAA GTA TAC CAG CCT-3’; *NEURL4*, forward 5’- CCG GAG GTG TAT GCA GTA GTC-3’, reverse 5’- AAC ACT GCC ACC GAG AAG TC-3’; *STON1*, forward 5’- TGG ATT ACT GAG GTG CCG TC-3’, reverse 5’- GCT TGC CTC TGA AAT GTG GC-3’. MANF 507: *TMEM107*, forward 5’- TAG TTG TCT GGC GGA AGG GA-3’, reverse 5’- AGC ACG TAA ATG ATC GGA GCA-3’; *AAGAB2*, forward 5’- AGA CTG ATT TCC CGC TCT GC-3’; reverse 5’- CAA GGA TAT ACT CCT GTA ACC TAG C-3’; *LRSPEI42*, forward 5’- AAC GTC CCT AAA ACC CGT CG-3’, reverse 5’-CCC CAG GAA AGC GGC ATT AT-3’. MANF 679: *DLG2*, forward 5’- GTT TTC CTC GCG TCT TGT GG-3’, reverse 5’- ACC TCT GGT AGC TGG GAA CT-3’; *MAX*, forward 5’- GTT TCA ATC TGC GGC TGA CA-3’, reverse 5’- CCA AGG AGA GAA GGC ATC CC-3’; *GLI4*, forward

5’- CTG GGA GTC AAC TTC GGT CG-3’, reverse 5’- TAC AAC TCG CTG CTG CTG AA-3’.

Generation of siRNA-resistant MANF plasmid

The MANF-HA plasmid was described in our previous study (Yang et al., 2017). To introduce point mutations that render plasmid resistant to MANF-siRNA, Uniclone One Step Seamless Cloning Kit (Genesand, SC612) was used following the manufacturer’s protocol. The primers used are listed as follows: forward 1, 5’-GCA CCT CGG TTC TAT CGA TTG AAT TCC GGA TGA GGA GGA TGT GGG CCA CG-3’; reverse 1, 5’-CTT AAG TTT CTC ACA AAT CTT TTC CAC AGG GAT GT-3’; forward 2, 5’-TGT GGA AAA GAT TTG TGA GAA ACT TAA GAA GAA GGA CA-3’; reverse 2, 5’-TGG AAC ATC GTA TGG ATA GCT GCC GCT GCC GCT GCC CAA ATC GGT CCG AGC ACT AGC TG-3’.

Statistical analysis

The data were analyzed by the Prism 9 (GraphPad) software. For comparisons between two groups, a two-tailed Student’s t-test was used. For three or more groups, one-way ANOVA with Tukey’s multiple comparisons tests was used. The quantitative results were presented as mean ± SEM. A P value < 0.05 was considered significant difference.

Availability of data and materials

All datasets generated and analyzed during this study and research materials are available from the corresponding author on reasonable request.

References

Yang, S., Yang, H., Chang, R., Yin, P., Yang, Y., Yang, W., Huang, S., Gaertig, M.A., Li, S., and Li, X.J. (2017). MANF regulates hypothalamic control of food intake and body weight. Nat Commun 8, 579.

Figure S1

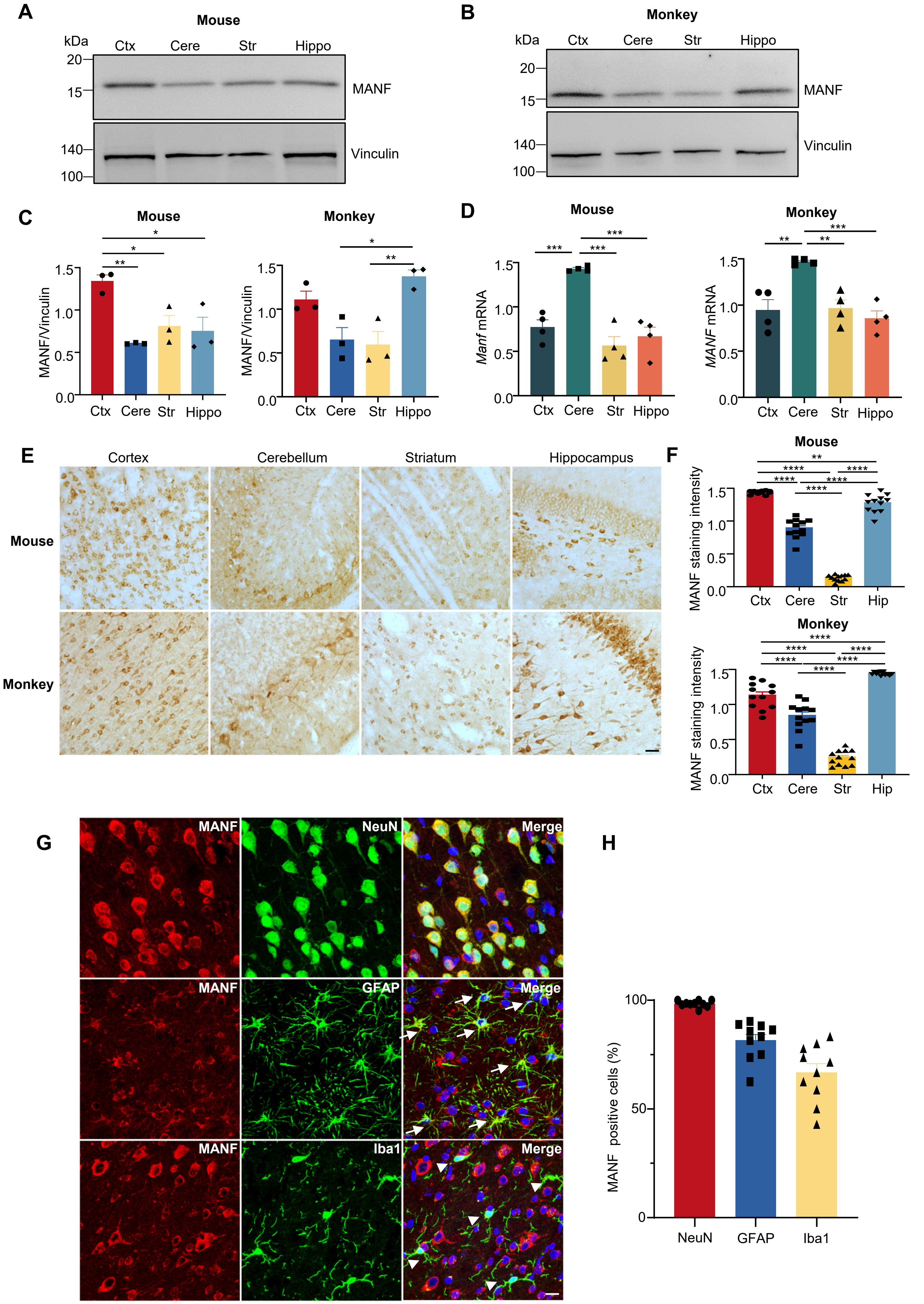

A
Mouse
kDa
Ctx
Cere
Str
Hippo
20
15
MANF
140
100
Vinculin
B
Monkey
kDa
Ctx
Cere
Str
Hippo
20
15
MANF
140
100
Vinculin
C
Mouse
Monkey
MANF/Vinculin
Ctx
Cere
Str
Hippo
D
Mouse
Monkey
Manf mRNA
MANF mRNA
Ctx
Cere
Str
Hippo
E
Cortex
Cerebellum
Striatum
Hippocampus
Mouse
Monkey
F
Mouse
Monkey
MANF staining intensity
Ctx
Cere
Str
Hip
G
MANF
NeuN
Merge
MANF
GFAP
Merge
MANF
Iba1
Merge
H
MANF positive cells (%)
NeuN
GFAP
Iba1

Figure legends

Figure S1. Characterization of MANF expression in the mouse and monkey brain.

(A) Western blotting analysis of MANF expression in different brain regions of the mouse brain (Ctx, cortex; Cere, cerebellum; Str, striatum; Hip, Hippocampus). **(B)** Western blotting analysis of MANF expression in different brain regions of the monkey brain. **(C)** Quantification of Western blotting results in Fig S1A and B (n = 3, one-way ANOVA with Tukey's post-tests). **(D)** Quantitative real-time PCR analysis of *Manf*/*MANF* mRNA in different brain regions of mice and monkeys (n = 4, one-way ANOVA with Tukey's post-tests). **(E)** Immunohistochemical staining of MANF in the mouse and monkey brain (scale bar: 50μm). **(F)** Quantification of MANF staining intensity in Fig S1E (n = 12, one-way ANOVA with Tukey's post-tests). **(G)** Double immunostaining of MANF with NeuN, GFAP, or Iba1 was performed on monkey brain slices (arrows indicate astrocytes with MANF staining; arrowheads indicate microglia with MANF staining; scale bar: 20μm). **(H)** Percentage of NeuN, GFAP and Iba1-positive cells that are MANF-positive (n = 10). * $P < 0.05$, ** $P < 0.01$, *** $P < 0.001$. Data is represented as mean ± SEM.

Figure S2

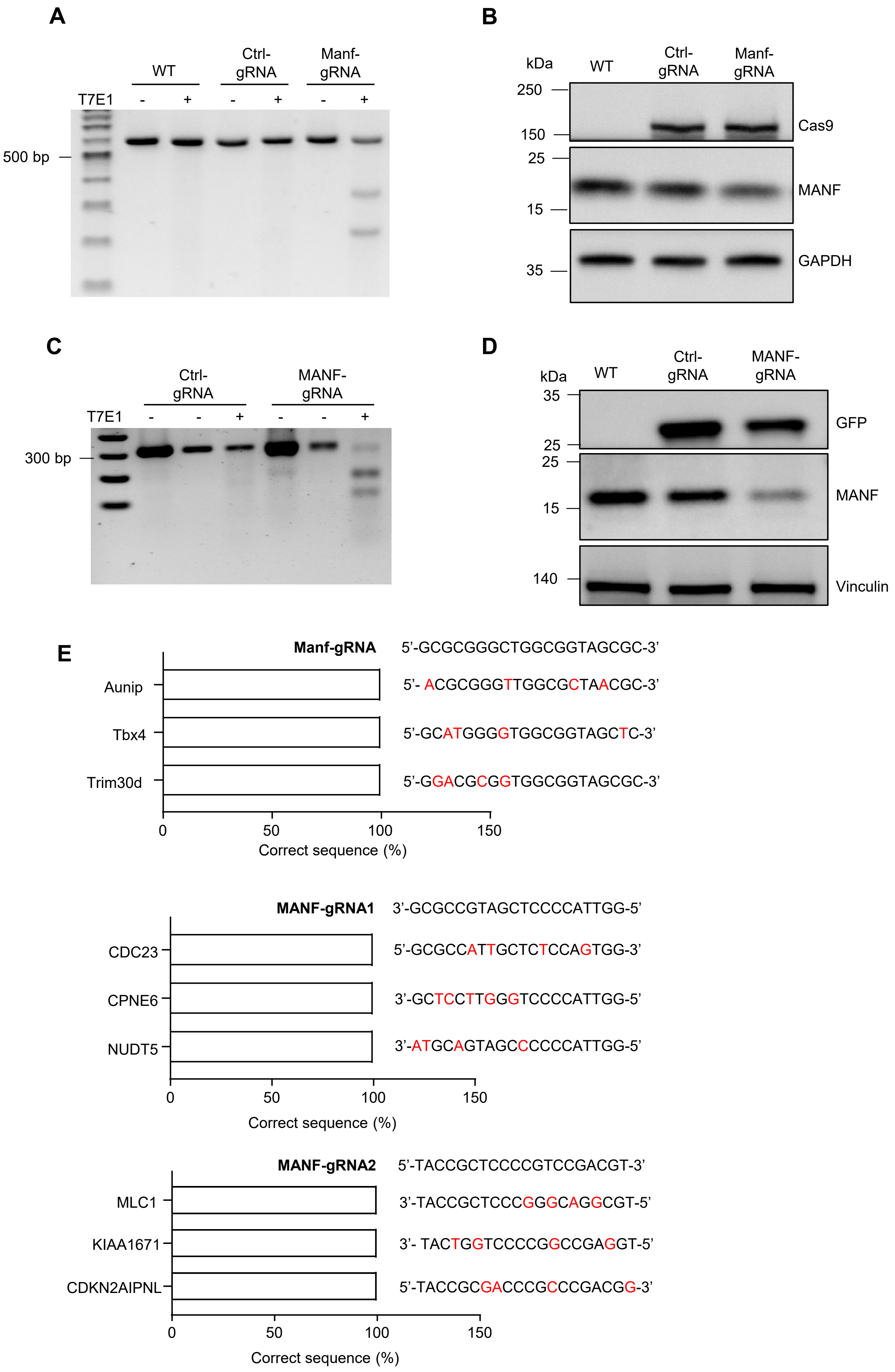
A
WT
Ctrl-gRNA
Manf-gRNA
T7E1
- + - + - +
500 bp
B
kDa
WT
Ctrl-gRNA
Manf-gRNA
250
150
25
15
35
Cas9
MANF
GAPDH
C
Ctrl-gRNA
MANF-gRNA
T7E1
- - + - - +
300 bp
D
kDa
WT
Ctrl-gRNA
MANF-gRNA
35
25
25
15
140
GFP
MANF
Vinculin
E
Manf-gRNA
5'-GCGCGGGCTGGCGGTAGCGC-3'
Aunip
5'- ACGCGGGTTGGCGCTAACGC-3'
Tbx4
5'-GCATGGGGTGGCGGTAGCTC-3'
Trim30d
5'-GGACGCGGTGGCGGTAGCGC-3'
0
50
100
150
Correct sequence (%)
MANF-gRNA1
3'-GCGCCGTAGCTCCCCATTGG-5'
CDC23
5'-GCGCCATTGCTCTCCAGTGG-3'
CPNE6
3'-GCTCCTTGGGTCCCCATTGG-5'
NUDT5
3'-ATGCAGTAGCCCCCCATTGG-5'
0
50
100
150
Correct sequence (%)
MANF-gRNA2
5'-TACCGCTCCCCGTCCGACGT-3'
MLC1
3'-TACCGCTCCCGGGCAGGCGT-5'
KIAA1671
3'- TACTGGTCCCCGGCCGAGGT-5'
CDKN2AIPNL
5'-TACCGCGACCCGCCCGACGG-3'
0
50
100
150
Correct sequence (%)

Figure S2 Verification of the genome editing efficiency of the selected gRNAs.

(A) T7E1 assay was performed using genomic DNAs extracted from N2A cells transfected with Cas9/Ctrl-gRNA plasmids or Cas9/Manf-gRNA plasmids. **(B)** Western blotting analysis of MANF in the N2A cells transfected with Cas9/Ctrl-gRNA plasmids or Cas9/Manf-gRNA plasmids. GAPDH was used as a loading control. **(C)** T7E1 assay was performed using genomic DNAs extracted from COS7 cells transfected with Cas9/Ctrl-gRNA plasmids or Cas9/MANF-gRNA plasmids. **(D)** Western blotting analysis of MANF in the COS7 cells transfected with Cas9/Ctrl-gRNA plasmids or Cas9/MANF-gRNA plasmids. Vinculin was used as a loading control. **(E)** The three most likely off-target loci of Manf-gRNA and MANF-gRNA were PCR-amplified and sequenced, and no mutations were detected at any of these sites.

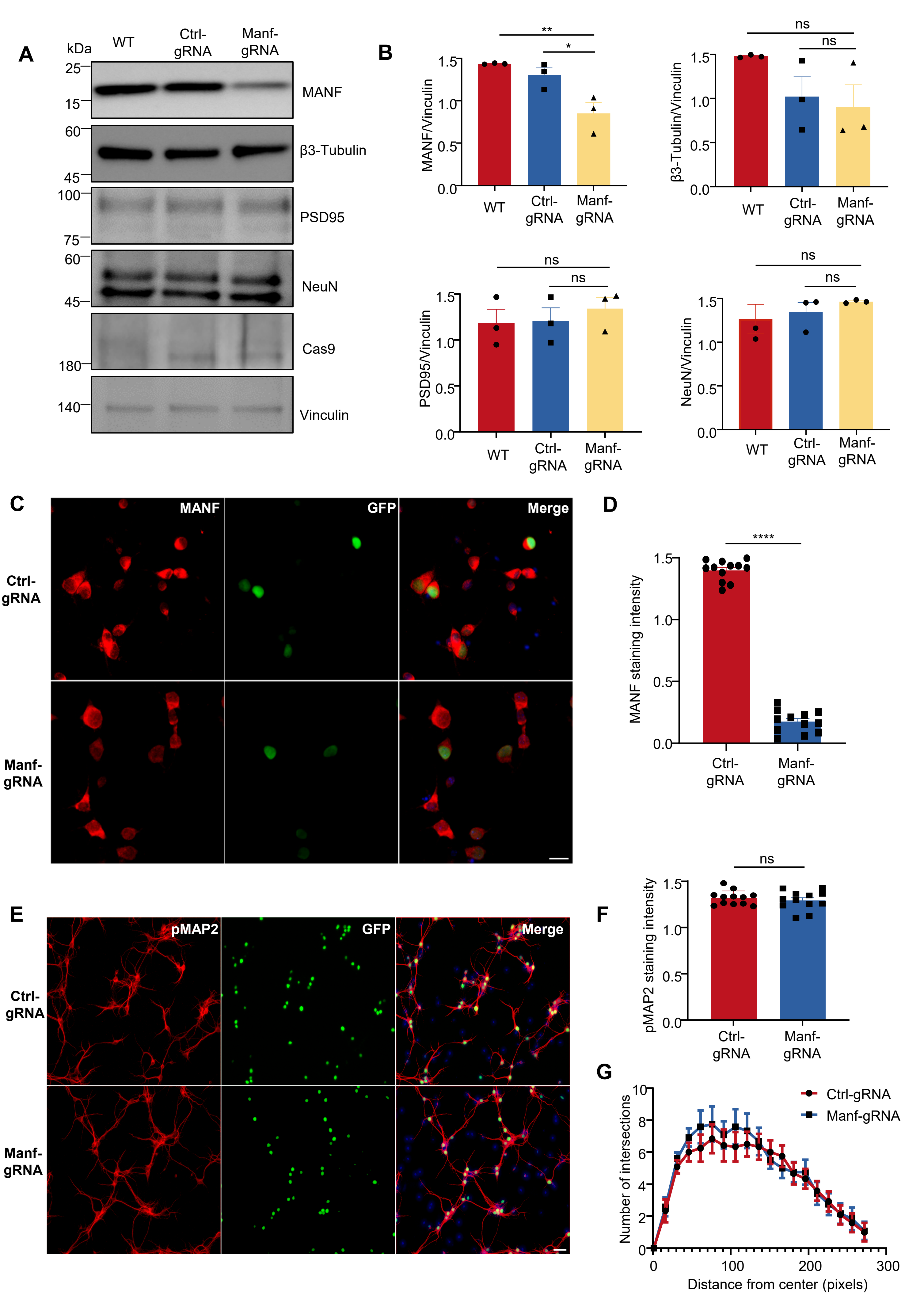
A
kDa
WT
Ctrl-gRNA
Manf-gRNA
25
15
MANF
60
45
β3-Tubulin
100
75
PSD95
60
45
NeuN
180
Cas9
140
Vinculin
B
MANF/Vinculin
**
*
β3-Tubulin/Vinculin
ns
ns
PSD95/Vinculin
NeuN/Vinculin
0.0
0.5
1.0
1.5
WT
Ctrl-gRNA
Manf-gRNA
C
MANF
GFP
Merge
Ctrl-gRNA
Manf-gRNA
D
MANF staining intensity
****
E
pMAP2
F
pMAP2 staining intensity
ns
G
Number of intersections
Distance from center (pixels)
0
100
200
300
2
4
6
8
10
Ctrl-gRNA
Manf-gRNA

Figure S3. Knockdown of MANF does not affect the survival of mouse primary neurons. **(A)** Western blotting analysis of MANF, β3-tubulin, PSD95, NeuN and Cas9 in the mouse primary neurons that were infected with either AAV-Cas9/AAV-Ctrl-gRNA or AAV-Cas9/AAV-Manf-gRNA. Vinculin was used as a loading control. **(B)** Quantification of western blotting results in Fig S3A (n = 3, one-way ANOVA with Tukey's post-tests). **(C)** Immunofluorescent staining of MANF in the mouse primary neurons that were infected with either AAV-Cas9/AAV-Ctrl-gRNA or AAV-Cas9/AAV-Manf-gRNA. GFP fluorescence reflected the virus-infected cells (scale bar: 20μm). **(D)** Quantification of MANF staining intensity in GFP-positive cells (n = 12, two-tailed Student's t-test). **(E)** Immunofluorescent staining of pMAP2 in the mouse primary neurons that were infected with either AAV-Cas9/AAV-Ctrl-gRNA or AAV-Cas9/AAV-Manf-gRNA (scale bar: 50μm). **(F)** Quantification of MANF staining intensity in Fig S3E (n = 12, two-tailed Student's t-test). **(G)** Sholl analysis of the distribution of dendritic intersections relative to the distance from the neuronal soma in Fig S3E (n = 12, two-way ANOVA with Sidak post-tests). Ns, non-significant, * $P < 0.05$, ** $P < 0.01$, **** $P < 0.0001$. Data is represented as mean ± SEM.

Figure S4

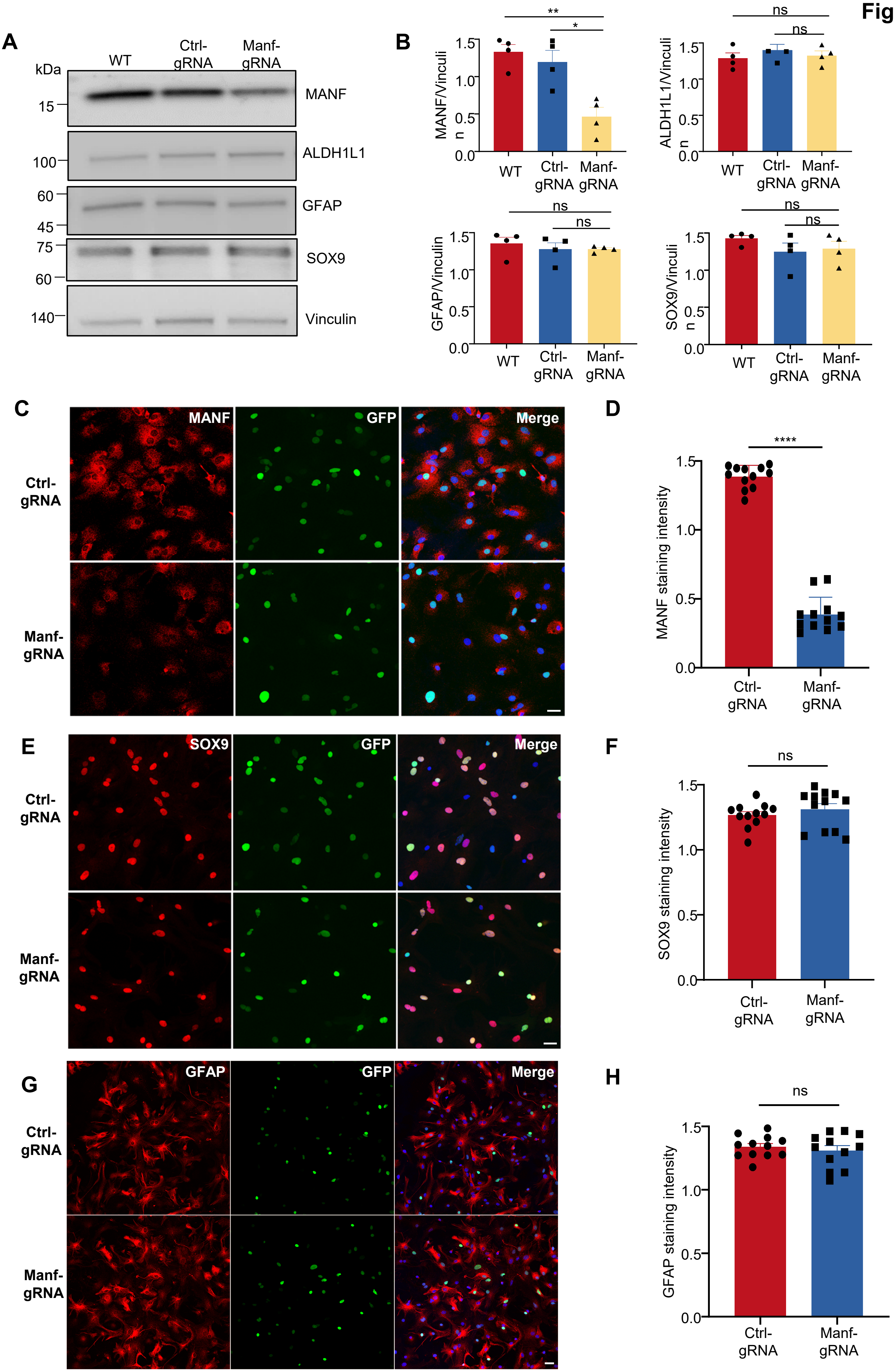

A
kDa
WT
Ctrl-gRNA
Manf-gRNA
15
MANF
100
ALDH1L1
60
45
GFAP
75
60
SOX9
140
Vinculin
B
MANF/Vinculi
ALDH1L1/Vinculi
GFAP/Vinculin
SOX9/Vinculi
**
*
ns
1.5
1.0
0.5
0.0
WT
Ctrl-gRNA
Manf-gRNA
C
MANF
GFP
Merge
Ctrl-gRNA
Manf-gRNA
D
****
MANF staining intensity
E
SOX9
F
SOX9 staining intensity
G
GFAP
H
GFAP staining intensity

Figure S4. Knockdown of MANF does not affect the survival of mouse primary astrocytes.

(A) Western blotting analysis of MANF, ALDH1L1, GFAP and SOX9 in the mouse primary astrocytes that were infected with either AAV-Cas9/AAV-Ctrl-gRNA or AAV-Cas9/AAV-Manf-gRNA. Vinculin was used as a loading control. **(B)** Quantification of western blotting results in Fig S4A (n = 4, one-way ANOVA with Tukey's post-tests). **(C)** Immunofluorescent staining of MANF in the mouse primary astrocytes that were infected with either AAV-Cas9/AAV-Ctrl-gRNA or AAV-Cas9/AAV-Manf-gRNA. GFP fluorescence reflected the virus-infected cells (scale bar: 20μm). **(D)** Quantification of MANF staining intensity in GFP-positive cells (n = 12, two-tailed Student's t-test). **(E)** Immunofluorescent staining of SOX9 in the mouse primary astrocytes that were infected with either AAV-Cas9/AAV-Ctrl-gRNA or AAV-Cas9/AAV-Manf-gRNA (scale bar: 20μm). **(F)** Quantification of SOX9 staining intensity in Fig S4E (n = 12, two-tailed Student's t-test). **(G)** Immunofluorescent staining of GFAP in the mouse primary astrocytes that were infected with either AAV-Cas9/AAV-Ctrl-gRNA or AAV-Cas9/AAV-Manf-gRNA. GFP fluorescence reflected the virus-infected cells (scale bar: 20μm). **(H)** Quantification of GFAP staining intensity in Fig S4G (n = 12 from three mice, two-tailed Student's t-test). Ns, non-significant, * $P < 0.05$, ** $P < 0.01$, **** $P < 0.0001$. Data is represented as mean ± SEM.

Figure S5

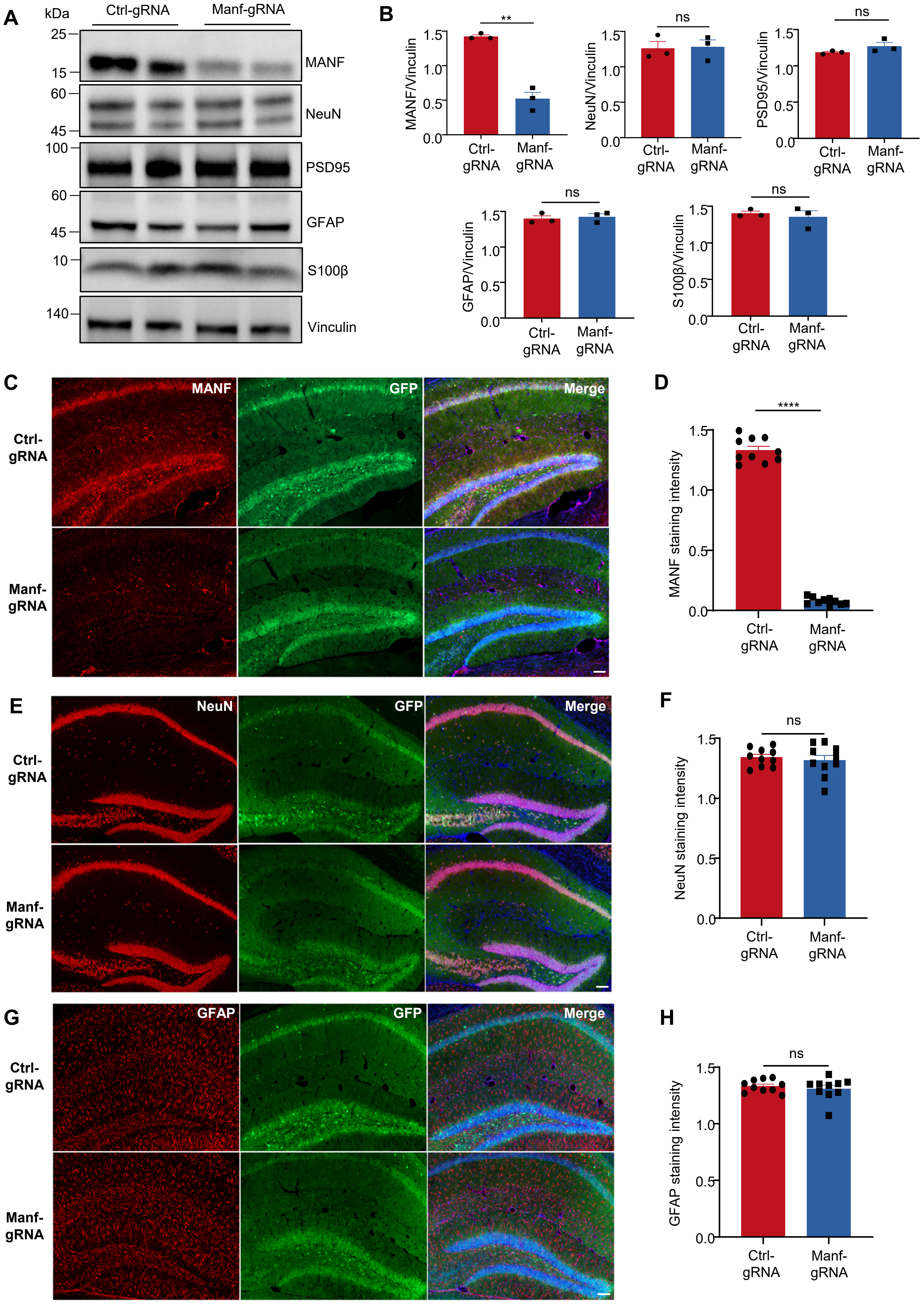
A
kDa
Ctrl-gRNA
Manf-gRNA
25
15
MANF
60
45
NeuN
100
PSD95
60
45
GFAP
10
S100β
140
Vinculin
B
MANF/Vinculin
**
NeuN/Vinculin
ns
PSD95/Vinculin
ns
GFAP/Vinculin
ns
S100β/Vinculin
ns
0.0
0.5
1.0
1.5
Ctrl-gRNA
Manf-gRNA
C
MANF
GFP
Merge
Ctrl-gRNA
Manf-gRNA
D
****
MANF staining intensity
E
NeuN
GFP
Merge
Ctrl-gRNA
Manf-gRNA
F
ns
NeuN staining intensity
G
GFAP
GFP
Merge
Ctrl-gRNA
Manf-gRNA
H
ns
GFAP staining intensity

Figure S5. Knockdown of MANF does not affect cell survival in the mouse hippocampus. **(A)** Western blotting analysis of MANF, NeuN, PSD95, GFAP and S100β in the hippocampus of WT mouse injected with AAV-Cas9/AAV-Ctrl-gRNA or AAV-Cas9/AAV-Manf-gRNA. Vinculin was used as a loading control. **(B)** Quantification of western blotting results in Fig S5A (n = 3, two-tailed Student's t-test). **(C)** Immunofluorescent staining of MANF in the hippocampus of WT mice injected with AAV-Cas9/AAV-Manf-gRNA or AAV-Cas9/AAV-Ctrl-gRNA. GFP fluorescence reflected the AAV infected cells (scale bar: 100μm). **(D)** Quantification of MANF staining intensity in Fig S5C (n = 10 from three mice, two-tailed Student's t-test). **(E)** Immunofluorescent staining of NeuN in the hippocampus of WT mice injected with AAV-Cas9/AAV-Manf-gRNA or AAV-Cas9/AAV-Ctrl-gRNA (scale bar: 100μm). **(F)** Quantification of NeuN staining intensity in Fig S5E (n = 10 from three mice, two-tailed Student's t-test). **(G)** Immunofluorescent staining of GFAP in the hippocampus of WT mice injected with AAV-Cas9/AAV-Manf-gRNA or AAV-Cas9/AAV-Ctrl-gRNA (scale bar: 100μm). **(H)** Quantification of GFAP staining intensity in Fig S5G (n = 10 from three mice, two-tailed Student's t-test). Ns, non-significant, ** $P < 0.01$, **** $P < 0.0001$. Data is represented as mean ± SEM.

Figure S6

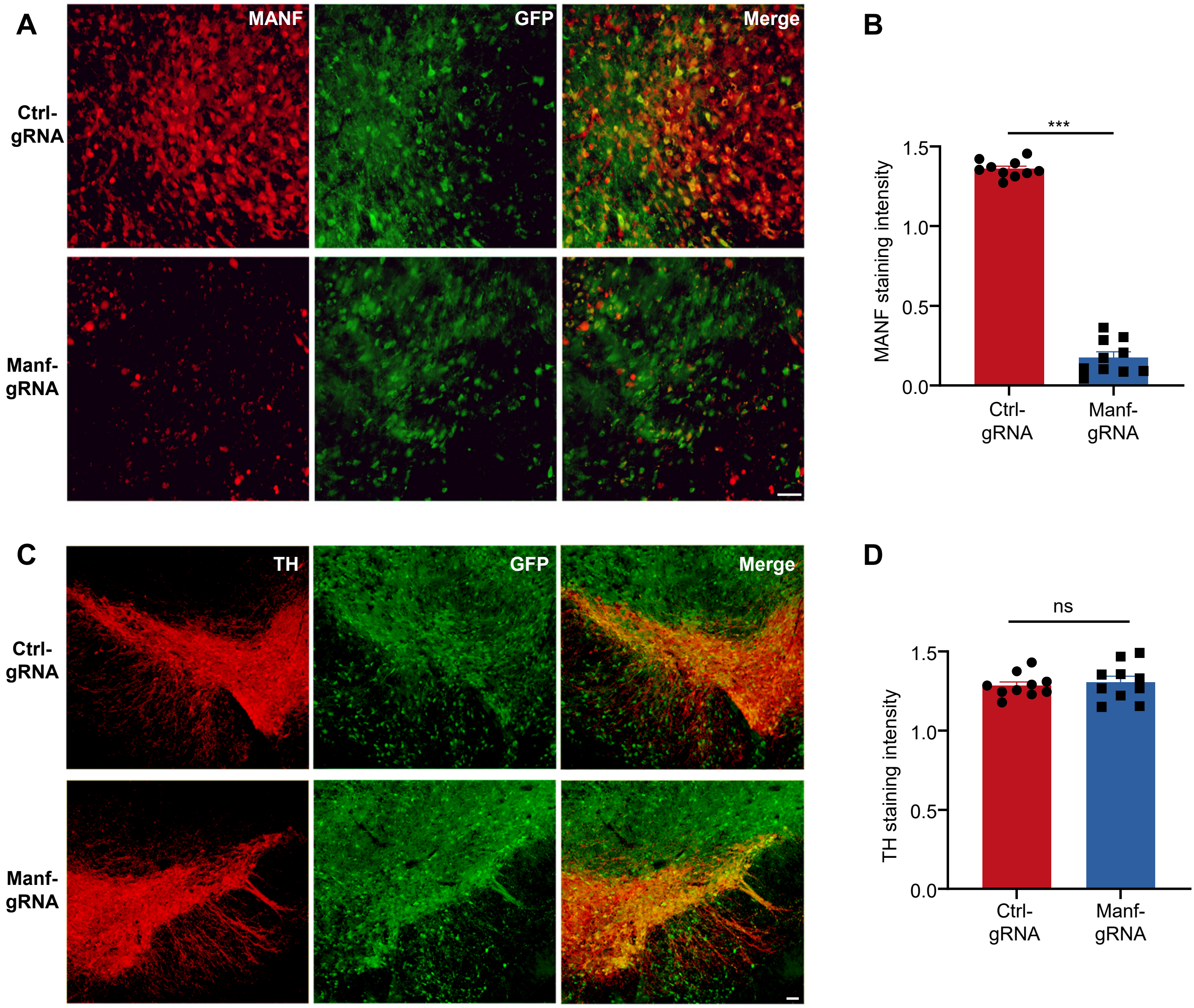

A
MANF
GFP
Merge
Ctrl-gRNA
Manf-gRNA
B
***
MANF staining intensity
1.5
1.0
0.5
0.0
Ctrl-gRNA
Manf-gRNA
C
TH
GFP
Merge
Ctrl-gRNA
Manf-gRNA
D
ns
TH staining intensity
1.5
1.0
0.5
0.0
Ctrl-gRNA
Manf-gRNA

Figure S6 Knockdown of MANF does not affect neuron survival in the mouse substantia nigra.

(A) Immunofluorescent staining images of MANF in the substantia nigra of WT mice injected with AAV-Cas9/AAV-Ctrl-gRNA or AAV-Cas9/AAV-Manf-gRNA (scale bar: 100μm). **(B)** Quantification of MANF staining intensity in Figure S6A (n = 10 from three mice, two-tailed Student's t-test). **(C)** Immunofluorescent staining images of TH in the substantia nigra of WT mice injected with AAV-Cas9/AAV-Ctrl-gRNA or AAV-Cas9/AAV-Manf-gRNA (scale bar: 100μm). **(D)** Quantification of TH staining intensity in Figure S6C (n = 10 from three mice, two-tailed Student's t-test). Ns, non-significant, *** $P < 0.001$. Data is represented as mean ± SEM.

Figure S7

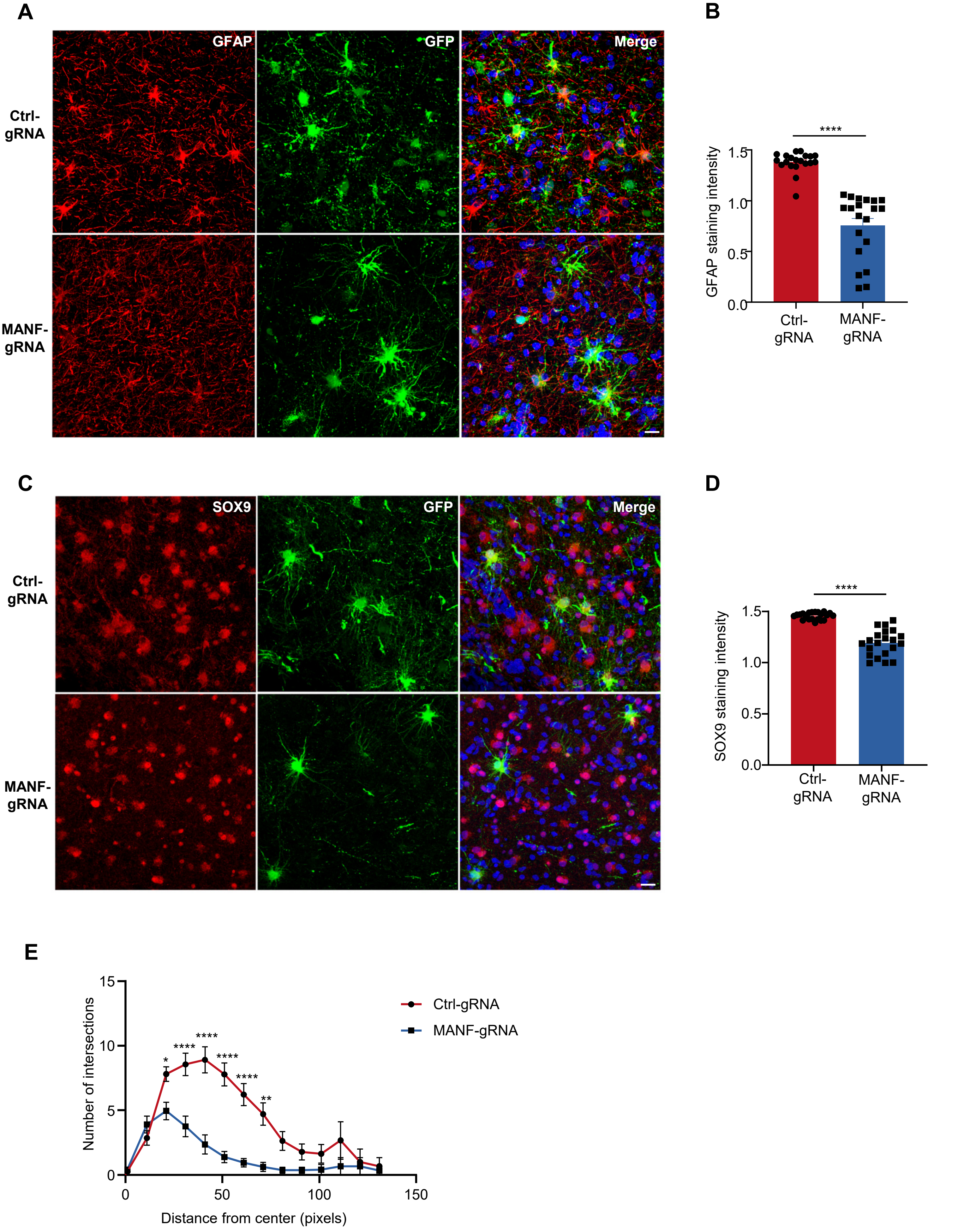
A
GFAP
GFP
Merge
Ctrl-gRNA
MANF-gRNA
B
GFAP staining intensity
****
1.5
1.0
0.5
0.0
Ctrl-gRNA
MANF-gRNA
C
SOX9
GFP
Merge
Ctrl-gRNA
MANF-gRNA
D
SOX9 staining intensity
****
1.5
1.0
0.5
0.0
Ctrl-gRNA
MANF-gRNA
E
Number of intersections
15
10
5
0
*
****
****
****
****
**
0
50
100
150
Distance from center (pixels)
Ctrl-gRNA
MANF-gRNA

Figure S7 Knockdown of MANF induces astrocyte loss in the monkey brain.

(A) Immunofluorescent staining images of GFAP in the brain of WT monkeys injected with AAV-Cas9/AAV-Ctrl-gRNA or AAV-Cas9/AAV-MANF-gRNA. GFP reflected the AAV infected cells (scale bar: 20μm). **(B)** Quantification of GFAP staining intensity in Figure S7A (n = 21 from three monkeys, two-tailed Student's t-test). **(C)** Immunofluorescent staining images of SOX9 in the brain of WT monkeys injected with AAV-Cas9/AAV-Ctrl-gRNA or AAV-Cas9/AAV-MANF-gRNA (scale bar: 20μm). **(D)** Quantification of SOX9 staining intensity in Figure S7C (n = 21 from three monkeys, two-tailed Student's t-test). **(E)** Sholl analysis of astrocyte process complexity using GFAP images in Fig S7A (n = 20 from three monkeys, two-way ANOVA with Sidak post-tests). * $P < 0.05$, ** $P < 0.01$, **** P < 0.0001. Data is represented as mean ± SEM.

Figure S8

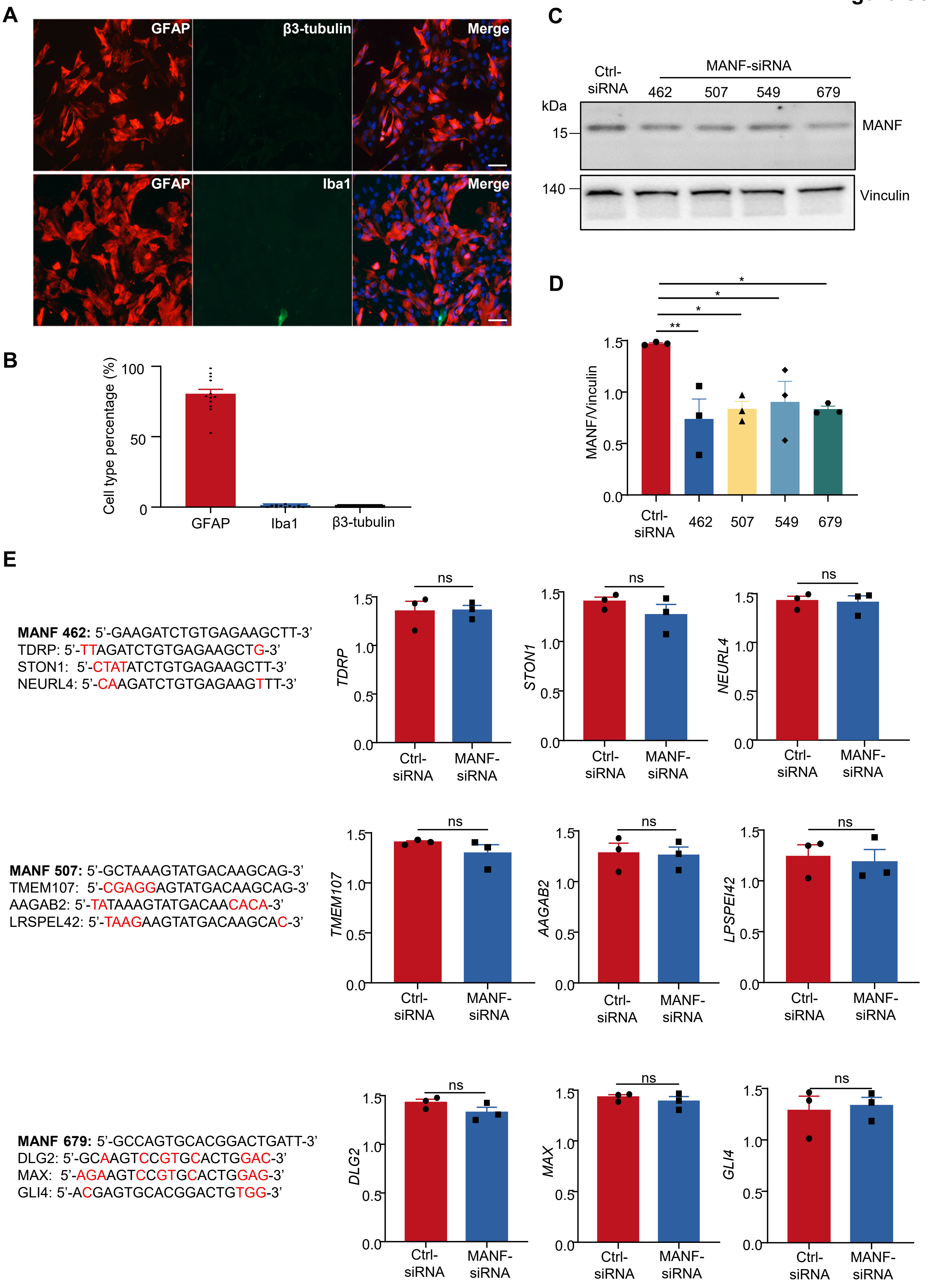
A
GFAP
β3-tubulin
Merge
GFAP
Iba1
Merge
B
Cell type percentage (%)
100
50
0
GFAP
Iba1
β3-tubulin
C
Ctrl-siRNA
MANF-siRNA
462
507
549
679
kDa
15
140
MANF
Vinculin
D
MANF/Vinculin
1.5
1.0
0.5
0.0
Ctrl-siRNA
462
507
549
679
**
*
*
*
E
MANF 462: 5'-GAAGATCTGTGAGAAGCTT-3'
TDRP: 5'-TTAGATCTGTGAGAAGCTG-3'
STON1: 5'-CTATATCTGTGAGAAGCTT-3'
NEURL4: 5'-CAAGATCTGTGAGAAGTTT-3'
MANF 507: 5'-GCTAAAGTATGACAAGCAG-3'
TMEM107: 5'-CGAGGAGTATGACAAGCAG-3'
AAGAB2: 5'-TATAAAGTATGACAACACA-3'
LRSPEL42: 5'-TAAGAAGTATGACAAGCAC-3'
MANF 679: 5'-GCCAGTGCACGGACTGATT-3'
DLG2: 5'-GCAAGTCCGTGCACTGGAC-3'
MAX: 5'-AGAAGTCCGTGCACTGGAG-3'
GLI4: 5'-ACGAGTGCACGGACTGTGG-3'
TDRP
STON1
NEURL4
TMEM107
AAGAB2
LPSPEI42
DLG2
MAX
GLI4
ns
Ctrl-siRNA
MANF-siRNA

Figure S8 Verification of monkey primary astrocyte culture and MANF-siRNA.

(A) Double immunofluorescent staining of monkey primary astrocytes using GFAP/β3-tubulin or GFAP/Iba1 antibodies (scale bar: 50μm). **(B)** Quantification of the percentage of GFAP, β3-tubulin and Iba1-positive cells relative to the total DAPI-positive nuclei in monkey primary astrocyte culture (n = 12). **(C)** Western blotting analysis of MANF in the COS7 cells transfected with Ctrl-siRNA or MANF-siRNA (462, 507, 549, 679), Vinculin was used as a loading control. **(D)** Quantification of western blotting results in Figure S8C (n = 3, one-way ANOVA with Tukey's post-tests). **(E)** The three most likely off-target transcripts of MANF-siRNA were analyzed using quantitative real-time PCR. Ns, non-significant, * $P < 0.05$, ** $P < 0.01$. Data is represented as mean ± SEM.

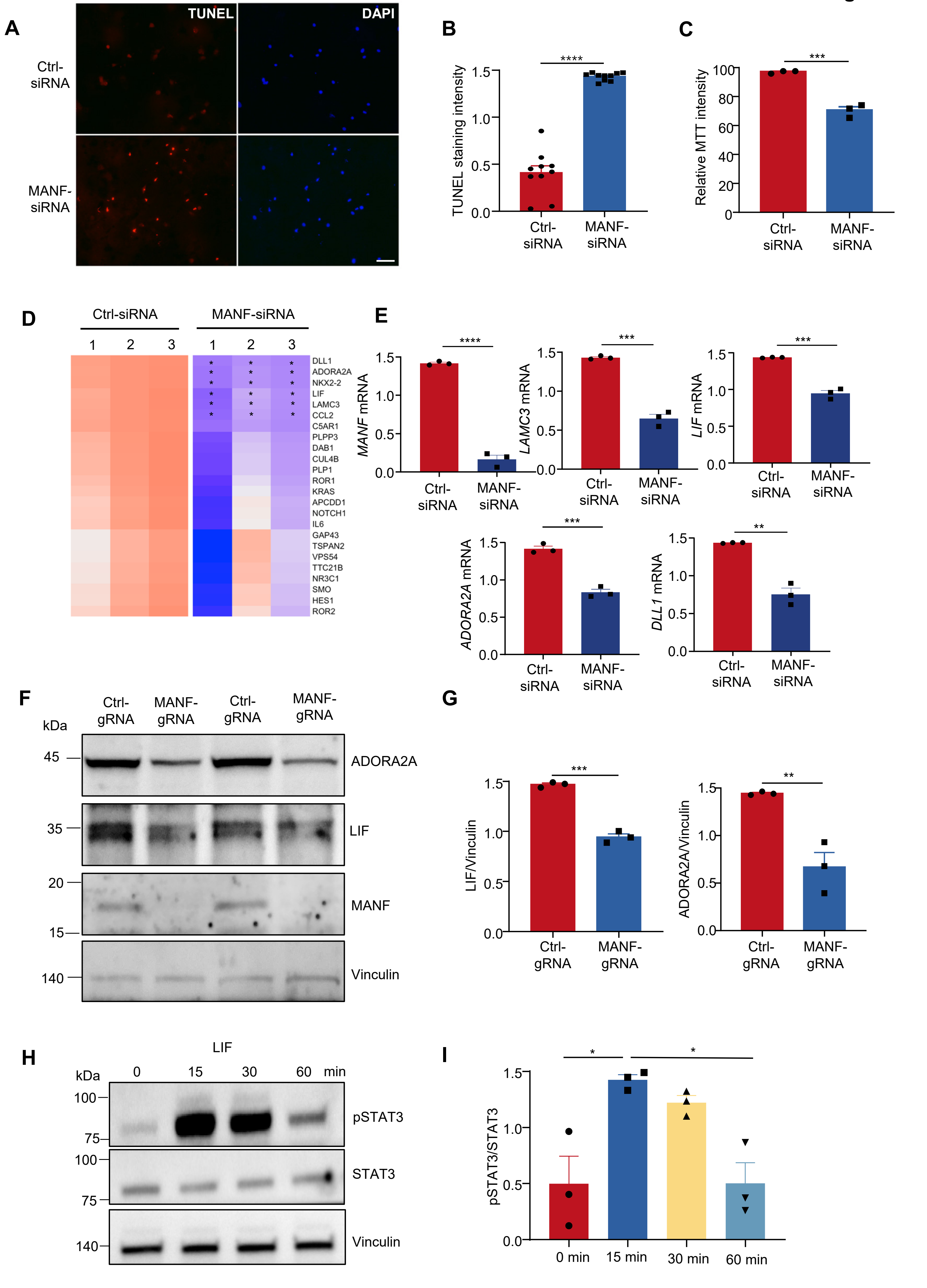
A
TUNEL
DAPI
Ctrl-siRNA
MANF-siRNA
B
TUNEL staining intensity
****
Ctrl-siRNA
MANF-siRNA
C
Relative MTT intensity
***
Ctrl-siRNA
MANF-siRNA
D
Ctrl-siRNA
MANF-siRNA
1 2 3
1 2 3
DLL1
ADORA2A
NKX2-2
LIF
LAMC3
CCL2
C5AR1
PLPP3
DAB1
CUL4B
PLP1
ROR1
KRAS
APCDD1
NOTCH1
IL6
GAP43
TSPAN2
VPS54
TTC21B
NR3C1
SMO
HES1
ROR2
E
MANF mRNA
LAMC3 mRNA
LIF mRNA
ADORA2A mRNA
DLL1 mRNA
Ctrl-siRNA
MANF-siRNA
F
kDa
Ctrl-gRNA
MANF-gRNA
Ctrl-gRNA
MANF-gRNA
45
35
20
15
140
ADORA2A
LIF
MANF
Vinculin
G
LIF/Vinculin
ADORA2A/Vinculin
Ctrl-gRNA
MANF-gRNA
H
LIF
kDa
0 15 30 60 min
100
75
100
75
140
pSTAT3
STAT3
Vinculin
I
pSTAT3/STAT3
0 min
15 min
30 min
60 min

Figure S9 knockdown of MANF affects LIF signaling in the monkey astrocytes.

(A) TUNEL staining images of monkey primary astrocytes that were electroporated with Ctrl-siRNA or MANF-siRNA (scale bar: 100μm). **(B)** Quantification of TUNEL staining intensity in Figure S9A (n = 10, two-tailed Student's t-test). **(C)** MTT assay evaluating the viability of primary astrocytes electroporated with Ctrl-siRNA or MANF-siRNA (n = 3, two-tailed Student's t-test). **(D)** Heatmaps showing the expression level of astrocyte marker genes when comparing the monkey primary astrocytes that were electroporated with Ctrl-siRNA or MANF-siRNA. **(E)** Quantitative real-time PCR analysis of the selected genes from the RNA-seq results (n = 3, two-tailed Student's t-test). **(F)** Western blotting analysis of MANF, LIF and ADORA2A in the brain of WT monkey injected with AAV-Cas9/AAV-Ctrl-gRNA or AAV-Cas9/AAV-MANF-gRNA. **(G)** Quantification of western blotting results in Fig S9F (n = 3, two-tailed Student's t-test). **(H)** Western blotting was used to analyze pSTAT3 and STAT3 in monkey primary astrocytes treated with 100 ng/ml LIF for 0 to 60 minutes. **(I)** Quantification of western blotting results in Figure S9H (n = 3, one-way ANOVA with Tukey's post-tests). Ns, non-significant, * $P < 0.05$, ** $P < 0.01$, *** $P < 0.001$, **** $P < 0.0001$. Data is represented as mean ± SEM.

Figure S10

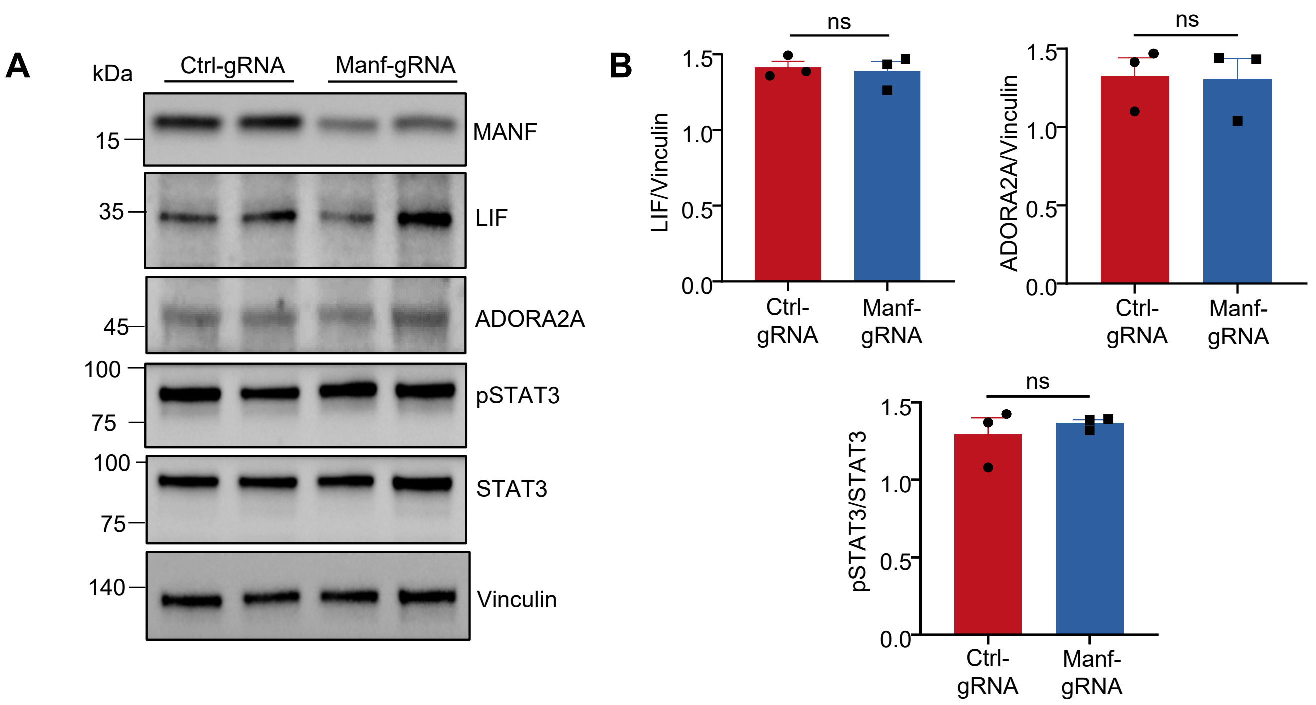
A
kDa
Ctrl-gRNA
Manf-gRNA
15
MANF
35
LIF
45
ADORA2A
100
75
pSTAT3
100
75
STAT3
140
Vinculin
B
ns
LIF/Vinculin
1.5
1.0
0.5
0.0
Ctrl-gRNA
Manf-gRNA
ns
ADORA2A/Vinculin
1.5
1.0
0.5
0.0
Ctrl-gRNA
Manf-gRNA
ns
pSTAT3/STAT3
1.5
1.0
0.5
0.0
Ctrl-gRNA
Manf-gRNA

Figure S10 Knockdown of MANF does not affect LIF-STAT3 pathway in the mouse primary astrocytes.

(A) Representative western blotting images of MANF, LIF, ADORA2A, pSTAT3 and STAT3 in the mouse primary astrocytes infected with either AAV-Cas9/AAV-Ctrl-gRNA or AAV-Cas9/AAV-Manf-gRNA. **(B)** Quantification of western blotting results in Figure S10B (n = 3, two-tailed Student's t-test). Ns, non-significant. Data is represented as mean ± SEM.
